# Root-Associated Microbial Communities of *Abies nordmanniana*: Insights Into Interactions of Microbial Communities With Antioxidative Enzymes and Plant Growth

**DOI:** 10.3389/fmicb.2019.01937

**Published:** 2019-08-22

**Authors:** Adriana M. Garcia-Lemos, Dominik K. Großkinsky, Michaela S. Stokholm, Ole S. Lund, Mette Haubjerg Nicolaisen, Thomas G. Roitsch, Bjarke Veierskov, Ole Nybroe

**Affiliations:** ^1^Department of Plant and Environmental Sciences, Faculty of Science, University of Copenhagen, Frederiksberg, Denmark; ^2^Copenhagen Plant Science Centre, Faculty of Science, University of Copenhagen, Frederiksberg, Denmark

**Keywords:** *Abies nordmanniana*, antioxidative enzymes, plant growth, root-associated microbial communities, *Rhizobiales*, *Agaricales*, *Fusarium*

## Abstract

*Abies nordmanniana* is a major Christmas tree species in Europe, but their uneven and prolonged growth slows down their production. By a 16S and 18S rRNA gene amplicon sequencing approach, we performed a characterization of root-associated bacterial and fungal communities for three-year-old *A. nordmanniana* plants collected from two nurseries in Denmark and Germany and displaying different growth patterns (small versus tall plants). Proteobacteria had the highest relative abundance at both sampling sites and plant sizes, and Ascomycota was the most abundant fungal phylum. At the order level, Acidobacteriales, Actinomycetales, Burkholderiales, Rhizobiales, and Xanthomonadales represented the bacterial core microbiome of *A. nordmanniana*, independently of the sampling site or plant size, while the fungal core microbiome included members of the Agaricales, Hypocreales, and Pezizales. Principal Coordinate Analysis indicated that both bacterial and fungal communities clustered according to the sampling site pointing to the significance of soil characteristics and climatic conditions for the composition of root-associated microbial communities. Major differences between communities from tall and small plants were a dominance of the potential pathogen *Fusarium* (Hypocreales) in the small plants from Germany, while Agaricales, that includes reported beneficial ectomycorrhizal fungi, dominated in the tall plants. An evaluation of plant root antioxidative enzyme profiles showed higher levels of the antioxidative enzymes ascorbate peroxidase, peroxidase, and superoxide dismutase in small plants compared to tall plants. We suggest that the higher antioxidative enzyme activities combined with the growth arrest phenotype indicate higher oxidative stress levels in the small plants. Additionally, the correlations between the relative abundances of specific taxa of the microbiome with the plant antioxidative enzyme profiles were established. The main result was that many more bacterial taxa correlated positively than negatively with one or more antioxidative enzyme activity. This may suggest that the ability of bacteria to increase plant antioxidative enzyme defenses is widespread.

## Introduction

The soil ecosystem contains the highest reported microbial diversity on earth (Torsvik et al., 2002) that serves as a resource wherefrom plant roots can recruit specific microbial communities to the rhizosphere (Mendes et al., 2013). The microorganisms in the rhizosphere can be beneficial, harmful or neutral for the growth and health of the plant. For example, plant growth promoting bacteria and fungi may enhance protection against pathogens, promote plant growth (Hardoim et al., 2015), and facilitate plant nutrition (Wu et al., 2015). In contrast, plant pathogenic bacteria and fungi represent a threat toward plant health (Plett and Martin, 2018). Plants and microbes interact, e.g., by signaling via root exudates. The composition of root exudates varies between plant species (Zhalnina et al., 2018), and this variability plays an important role for the establishment of plant-rhizospheric microbial communities (Bais et al., 2006; Chaparro et al., 2013).

*Abies nordmanniana* (Steven) Spach is a major Christmas tree species grown throughout Europe, where the production exceeds 30 million trees annually (Bräuner Nielsen et al., 2011). However, the natural growth of this species is slow, and trees only reach commercial use after several years. The ability of *A. nordmanniana* to select specific rhizosphere microbial communities has so far not been addressed; nor have these communities been characterized, although they represent a possible source for plant growth and health promoting microorganisms. Many conifer species develop associations with beneficial microorganisms; in particular ectomycorrhizal fungi (EM), which enhance plant nutrition and growth (Marx, 1970; Ważny, 2014; Rudawska et al., 2016). Similarly, the study of Zulueta-Rodriguez et al. (2015) demonstrated positive effects of seed inoculation with known plant growth promoting rhizobacteria (*Bacillus subtilis, Pseudomonas fluorescens, and P. putida*), previously isolated from other plant species. These inoculants enhance germination and seedling growth of *A. hickelii* and *A. religiosa;* two endangered Mexican pine species. On the other hand, several fungal pathogens, e.g., from the genera *Armillaria*, *Fusarium*, and *Rhizoctonia* can negatively influence the health of *Abies* species (Oliva et al., 2009; Gordon et al., 2015), while EM in symbiosis with *Abies* may even offer protection against pathogens in seedlings (Ważny, 2014).

In plants, environmental stressors such as drought and high salinity lead to the accumulation of reactive oxygen species (ROS), which can cause severe damage to the cells (Caverzan et al., 2016). To protect themselves, plants launch an antioxidative defense, where antioxidant (ROS scavenging) enzymes play a major role (Gill and Tuteja, 2010). Hence, activities of enzymes such as catalase (CAT), superoxide dismutase (SOD) ascorbate peroxidase (APX) and peroxidase (POX) often increase under abiotic stress (Mhadhbi et al., 2004; Jebara et al., 2005; Kohler et al., 2008; Mandal et al., 2008; Bharti et al., 2016; Caverzan et al., 2016; Sarkar et al., 2018). In addition to their role in coping with ROS formation caused by environmental stresses, antioxidant enzymes are essential for the maintenance of cell redox homeostasis. By regulating ROS deriving from central processes in different cellular compartments such as photosynthesis and respiration, they can contribute to the regulation of plant growth processes (Das et al., 2015).

Microorganisms introduced to the rhizosphere can affect the activity of antioxidative defense pathways in plants. Hence, Bharti et al. (2016) reported that the expression of several defense enzymes was enhanced by an introduced rhizobacterium in wheat under salt stress, and thereby mediated salinity tolerance. However, there are even examples of introduced strains mediating drought tolerance that decrease the levels of antioxidative enzymes in plants (Sandhya et al., 2010; Armada et al., 2016). Moreover, it has been suggested that fungal endophytes might contribute to redox regulation, improving the antioxidant capacity of the plant host by regulating the host genes. Hence, Brotman et al. (2013) reported that *Trichoderma* induced expression of genes coding for antioxidant enzymes in *Arabidopsis thaliana.* While the effects of specific, introduced microorganisms on antioxidative plant enzymes have been assessed in inoculated plants and *in vitro* studies, these interactions, to the best of our knowledge are poorly understood in field-grown plants interacting with natural root-associated microbial assemblages. The different results, however, point at different mechanisms whereby microbes mediate beneficial effects to plants, ranging from stress reduction and avoidance to improved stress tolerance.

A deeper insight into the composition of microbial communities associated with *A. nordmanniana* and their interactions with plant antioxidative enzymes, and ultimately plant growth phenotype, is highly relevant to facilitate strategies for microbial mediated growth promotion of *A. nordmanniana*. In this study, we characterized and compared the composition of root-associated bacterial and fungal communities of *A. nordmanniana* showing different growth habits when grown at two field sites. In parallel, we determined signatures of *A. nordmanniana* antioxidative enzyme activities in roots. Finally, we established correlations between the relative abundances of specific microbial taxa and the plant antioxidative enzyme profile in the context of the tree growth phenotype.

## Materials and Methods

### Sampling Site

Seedlings of *A. nordmanniana* were collected from two Christmas tree nurseries; sampling site 1: Baumschule Engler located in Hohenlockstedt, Germany (53°58′30.5′′N 9°37′51.9′′E), and sampling site 2: Primo Plant Ejendomme ApS located in Hadsund, Denmark (56°44′22.7′′N 10°03′36.7′′E). Sampling at both sites was performed during July 2016, the climatic conditions for both sampling sites were accessed using the German National meteorological Service^1^ and the Danish National Meteorological Service^2^. During the sampling dates, the Danish site presented a higher maximum temperature of 28°C, and the German site presented a maximum temperature of 21°C (Supplementary Table S1). The German site presented the higher variation in rainfall during the 3 months prior to sampling, with values between 21 and 101 mm, and at the Danish site, the differences in rainfall ranged between 41 and 80 mm (Supplementary Table S1).

Additionally, the climatic data for both sampling sites was also accessed for the 3 years of growth of the sampled *A. nordmanniana* plants. The yearly average temperature showed comparable maximum and minimum temperatures, and comparable yearly hours of sun for the German and Danish sampling sites, respectively (Supplementary Table S1). However, higher average rainfall values were observed for the Danish site (932 mm) during the 3-year period, compared with the German site (801 mm) (Supplementary Table S1).

### Plant Material

Christmas tree production in each nursery derived from the same parental seed source: Berritzgaard F 665, which originates from a small area in Ambrolauri, Georgia. According to the seed providers Levinsen and Abies A/S, the second generation of Berritzgaard plants was raised at the Hildesvig forest in Denmark. Since 1991, the trees at the Berritzgaard plantation have been selected for uniformity in traits of importance for Christmas tree production.

*Abies nordmanniana* plants (3 years old) displaying different growth were collected at each sampling site, where five plants with an average height of 25 cm, hereafter referred to as “small” plants, and five plants with an average height of 60 cm, hereafter referred to as “tall” plants, were sampled (Supplementary Table S2). Each plant was considered as a biological replicate for plant size and sampling site. Plant heights were measured from the top of the apical bud to the end of the main root. The plants were collected in their entirety with the surrounding bulk soil and transferred into individual pots, in which they remained for 1.5 days at 18°C in a greenhouse, at the Department of Plant and Environmental Sciences, University of Copenhagen, Frederiksberg, Denmark, before samples for DNA extraction, plant enzyme analysis and soil analysis were taken.

### Soil Characterization

In order to perform soil characterization, three soil sub-samples from each sampling site were randomly collected with a hand shovel and represented top soil down to 30 cm depth. Soil samples were placed in plastic bags and later combined per sampling site. For soil analysis, 500 g samples were used; soil physico-chemical analyses included the determination of concentrations of macro and micronutrients, soil organic matter and soil texture. The analyses were performed using ISO certified procedures at Eurofins Agro Testing, Denmark, and method specifications can be found at www.eurofins.dk. Elemental analyses employed Inductively Coupled Plasma – Optical Emission Spectrometry (ICP-OES), or Flow Injection Analysis- spectrometry (FIA) as specified in Supplementary Table S3. Additionally, soil pH was measured by following the protocol of Hanlon (2012).

### Rhizosphere and Bulk Soil DNA Extraction

Plants were removed from the pots and the bulk soil was removed from the roots with a brush. Subsequently, entire root systems with primary and secondary roots (∼5–10 mm diameter and ∼15–20 cm length) with firmly adhering soil were cut into short fragments with a sterile scalpel. The fragments were then macerated with liquid nitrogen in a mortar, to obtain finely ground and well-mixed samples representing plant tissue and rhizosphere soil. These root-associated samples are hereafter referred to as rhizosphere samples.

For each plant, 0.25 g of rhizosphere and bulk soil samples were used for DNA extraction, which was carried out using the PowerSoil^®^ DNA Isolation Kit (Mo Bio Laboratories, Carlsbad, CA, United States) according to the manufacturer’s instructions. The nucleic acid concentration of each sample was quantified using a NanoDrop 2000c spectrophotometer (Thermo Fisher Scientific, Wilmington, DE, United States) (Desjardins and Conklin, 2010).

### Enzyme Activity Signatures of Plant Antioxidative Metabolism

After the recovery of plant material with firmly adhering rhizosphere soil for DNA extraction, the remaining root tissue was washed with tap water to remove any remaining soil particles and was used for the determination of plant physiological parameters. For these determinations, root material was snap frozen and ground in liquid nitrogen and stored at −80°C until further processing.

To determine activity signatures of enzymes in the plant antioxidative system, proteins were extracted from root samples according to LaFever et al. (1994) with few adjustments. Briefly, 100 mg ground material was mixed with 100 mg Amberlite XAD-4 and 100 mg PVPP and was extracted with 1.5 ml extraction buffer (100 mM potassium phosphate buffer, pH 7.0, 5 mM ascorbate, 5 mM DTT, 5 mM sodium bisulfite, 7.5 mM MgCl_2_, 20 μM MnCl_2_, 10% glycerol, 1% PVP; LaFever et al., 1994). Cell wall-bound proteins were extracted from the remaining pellet with a high-salt buffer (1 mM NaCl, 40 mM Tris–HCl, pH 7.6, 3 mM MgCl_2_, 1 mM EDTA, 0.1 mM PMSF, 1 mM benzamidine, 14 mM β-mercaptoethanol, 24 μM NADP) according to Jammer et al. (2015).

Activities of the antioxidative enzymes APX, CAT, cell wall and cytoplasmic peroxidases (cwPOX, POX), glutathione reductase (GR) and SOD were determined photometrically by kinetic assays in a miniaturized 96-well plate format following the approach presented in Jammer et al. (2015). The assays were based on principles published by Yoshimura et al. (2000) for APX, Aebi (1984) for CAT, Polle et al. (1994) for (cwPOX) POX, Edwards et al. (1990) for GR and McCord and Fridovich (1969) for SOD.

### 16S rRNA Gene Amplicon Sequencing

To confirm the presence of bacterial DNA in the rhizosphere and bulk soil samples, the 16S rRNA gene region was amplified by PCR using the following protocol; 2 μl of extracted DNA was added to a PCR reaction mix containing 10× PCR buffer without salt (Sigma-Aldrich Co.), 25 mM of MgCl_2_ (Sigma-Aldrich Co.), 10 mM of each dNTP, 5 U Taq DNA polymerase (Sigma-Aldrich Co.), 10 μM of 341 Forward primer and 10 μM of 806 Reverse primer [primer sequences are in Sundberg et al. (2013)] and molecular grade water (Sigma-Aldrich Co.) up to a final volume of 50 μl. Primers were obtained from TAG Copenhagen A/S, Copenhagen, Denmark. The PCR amplification was conducted in a Mastercycler Pro thermal cycler (Eppendorf, Hauppauge, NY, United States), as follows: an initial denaturation step consisting of 4 min at 94°C; 35 cycles of 30 s at 94°C, 1 min at 55°C, 1 min at 72°C, and a final extension of 7 min at 72°C. The PCR products were visualized using a gel documentation system (Bio-Rad Gel Doc^TM^ 2000 Bio-Rad Laboratories CO) after agarose gel electrophoresis and gel staining with GelRed^TM^ Nucleic Acid Gel Stain (Biotium Inc., United States).

DNA samples validated by the above PCR assay were sent to Macrogen Inc. (Gasan-dong, Seoul, South Korea), for amplicon sequencing of the V3-V4 regions of the 16S rRNA gene using the primers Bakt 341F and Bakt 805R (Herlemann et al., 2011). Sequencing was performed using the Illumina MiSeq system (Paired-end, 2 × 301 bp, Macrogen, Gasan-dong, Seoul, South Korea).

Sequencing data was imported to CLC Genomics Workbench Version 8.0^3^. The raw data was processed following the CLC Microbial Genomics Module pipeline. Briefly, sequences were paired, then, the primer sequences were trimmed off with the following parameters: removal of low-quality sequence (limit = 0.05), removal of ambiguous nucleotides: maximal two nucleotides allowed. The removal of adapter sequences was performed using the following adapters: Bakt_805R (GACTACHVGGGTATCTAATCC), strand = Plus, action = Remove adapter, score = [2: 3: 8: 8], and Bakt_341F (CCTACGGGNGGCWGCAG), strand = Plus, action = Remove adapter, score = [2: 3: 8: 8], removal of sequences on length: minimum length five nucleotides. Next, the paired read sequences were merged, and the length was fixed by trimming to an exact length of 402 bp.

Samples were filtered based on the number of reads, with a minimum number of reads set to 100, discarding the samples that did not fulfill this parameter. Subsequently, the filtered sequences with comparable length and coverage were clustered and assigned to operational taxonomic units (OTUs). A 97% of similarity was used when comparing with the Greengenes 16S rRNA gene database. A list of sequences that clustered with sequences in the Greengenes database was obtained and used to generate the abundance OTU table. Furthermore, chimeras were detected and removed in this step. Subsequently, an OTU relative abundance table was generated by summing up all OTU counts and dividing each count with the total sum.

### 18S rRNA Gene Amplification and Sequencing

Isolated DNA from rhizosphere samples was PCR amplified using one forward and two reverse primers for multiplex PCR targeting two universally conserved domains. The assay amplified a variable region (of ∼400 bp) from the small subunit of 18S rRNA gene of plant-associated microbial eukaryotes: basidiomycetes, ascomycetes, glomeromycetes, zygomycetes, oomycetes, plasmodiophorids and nematodes. Primers were modified after Stokholm et al. (2016) to match the requirements for amplicon sequencing using the Illumina MiSeq platform. The 18S rRNA gene reverse primers contain at least one mismatch relative to sequence accessions representing *Pinaceae* species. Primers were obtained from Eurofins Genomics, Ebersberg, Germany. The forward primers were fused to a sequencing linker overhang, followed by 10 bp different internal multiplex identifier (MID), tags for barcoding of different samples, 2 × NN, and a variable heterogeneity spacer from 0 to 7 bp to generate primers of different lengths according to Fadrosh et al. (2014; Supplementary Table S4). The reverse primers consisted of a linker overhang, followed by internal MID tags, 2 × NN and a 12 bp insert in front of the reverse primer.

The 18S rRNA gene-targeted multiplex PCR reaction (1×), consisted of 20 ng DNA, 1.5 μM 18S forward primer 18S-F2-LIM, 2.25 μM reverse primer 18S-R2aeh-LIM, 0.75 μM reverse primer 18S-R2g-LIM, 1.5 mM MgCl_2_, 1× colorless GoTaq Reaction buffer, 24 μU GoTaq DNA Polymerase (Promega Corporation, WI, United States), 0.2 mM dNTP, 2.5% DMSO and H_2_O up to a final volume of 50 μl. PCR amplification was conducted in a thermal cycler (MJ mini^TM^ Personal Thermal Cycler, Bio-Rad Laboratories Inc.), with the following program: 3 min initial DNA denaturation at 95°C, followed by a phase one of 12 cycles of 20 s at 94°C, 20 s at 50°C, 30 s at 72°C and a phase two of 32 cycles of 20 s at 94°C, 20 s at 65°C, 30 s at 72°C and a 7 min final extension at 72°C. PCR products were assessed by gel electrophoresis in 1.5% agarose gels. 18S rRNA gene amplicons, ∼520 bp for 18S rDNA were excised from gels and purified using MinElute Gel Extraction Kit (Qiagen Group, CA). DNA concentrations of the purified amplicons were determined using a NanoDrop ND-100 spectrophotometer, according to the manufacturer’s specifications (NanoDrop Technologies Inc., Wilmington, DE, United States).

Equimolar concentrations of the 18S rRNA gene amplicons, were mixed and sequenced in parallel at the Illumina MiSeq platform at the Danish Technological Institute, Department of Food Security, Taastrup, Denmark. Next a de-multiplexing of the sequences using sabre (a barcode de-multiplexing and trimming tool for FastQ files^4^) was made in a virtual Linux environment, according to the internal MID tags. The resulting de-multiplexed paired-end FastQ files were then individually overlap-merged and quality-filtered through a series of sequence trimming and quality control (QC) steps. The quality filtered datasets were then subjected to the open source SCATA pipeline (Sequence Clustering and Analysis of Tagged Amplicons^5^) at the Swedish University of Agricultural Sciences (Clemmensen et al., 2016), for single linkage clustering into OTUs, using a 98% similarity cut-off and parameters as described in Stokholm et al. (2016). The representative sequences of the OTUs were further analyzed and annotated down to the order level, or to the lowest possible taxonomic level, by comparison to similar sequence accessions in GenBank^6^, as well as by comparison, where possible, to sequences in the SILVA SSU database for 18S sequences^7^ (Yilmaz et al., 2014).

### Data Analysis

The bacterial and eukaryotic OTU relative abundance tables were used to estimate OTU diversity using PAST version (2.17). The α-diversity, represented by Shannon H and Chao-1 estimations, was calculated in PAST and compared by Student’s *t*-test. The dissimilarity of the microbial communities from bulk soil (bacterial community only) and rhizosphere samples from the two sampling sites, and for small versus tall plants was calculated using the Bray–Curtis index.

The differences in taxa abundance at the phylum level between the sampling sites and plant sizes were compared by Student’s *t*-test using PAST version (2.17). Dissimilarities of the relative abundance to determinate the microbial taxa at order and genus level contributing for the dissimilarity among sampling sites were calculated using the similarity percentage analysis (SIMPER) with the Bray–Curtis dissimilarity index in PAST version (2.17). With the SIMPER analysis, the average Bray–Curtis dissimilarities between all analyzed groups (sampling site and plant size) is decomposed into percentage of contributions of each OTU filtered at the order level, giving the list in decreasing order of contribution of dissimilarity (Clarke and Gorley, 2006). The SIMPER analysis should be considered carefully for taxa with a high variance, as it can fail to properly identify taxa with large between group effects (Warton et al., 2012). In the current analyses, the variances for the taxa contributing to variability between treatments were low; therefore, we have chosen to use SIMPER for identifying the taxa contributing to differences between sampling sites and plant sizes.

Principal Coordinate Analysis (PCoA), based on the Bray–Curtis dissimilarity matrix between rhizosphere samples were performed with the Phyloseq R package version (2.4-3). A permutational multivariate analysis of variance (Adonis), based on the Bray–Curtis distances with 9999 permutations, was used to access the statistical significance of the microbial community between sampling sites, bulk soil versus rhizosphere samples and tall versus small plants. Additionally, a Canonical Correspondence Analysis (CCA) was performed using PAST version (2.17), including the data from the antioxidative enzymatic activities of each root sample, with the bacterial and fungal OTU relative abundance tables filtered at the order level for bacterial communities and order and genus (for Hypocreales) level for fungal communities. Additionally, a one-way ANOVA was used to test the effect of plant size on the fungal relative abundance at order level. Further, the correlation statistic according to Spearman was used to test the significant correlations between the enzymatic variables and the OTU tables at the order or genus level.

Venn diagrams were constructed using the software Venny (Oliveros, 2006), to determinate the unique and shared OTUs between each sampling site and plant size. For this, the filtered list of OTUs at the order level for both bacterial and fungal communities, were used. For each constructed Venn diagram, the mean relative abundance of the shared and unique OTUs at the order level was used to construct stacked bar plots.

The 16S and 18S rRNA gene amplicon sequences were deposited in NCBI’s Sequence Read Archive under BioProject PRJNA515250.

## Results

### Soil Characteristics

Soil from the two sampling sites were both sandy loams with 5–7% clay and 84–90% sand. Overall soil from both sampling sites presented comparable concentrations of nitrogen and organic matter (Supplementary Table S3). More than two-fold differences in chemical composition were observed for manganese and boron content, which was higher in soil derived from the sampling site in Germany compared with the sampling site in Denmark. Danish bulk soil samples had higher amounts of potassium, magnesium, copper and iron (Supplementary Table S3). Soil pH values were comparable, with values of 5.8 and 5.5 for Germany and Denmark, respectively.

### Root Antioxidative Enzyme Activity Signatures

**FIGURE 1:**
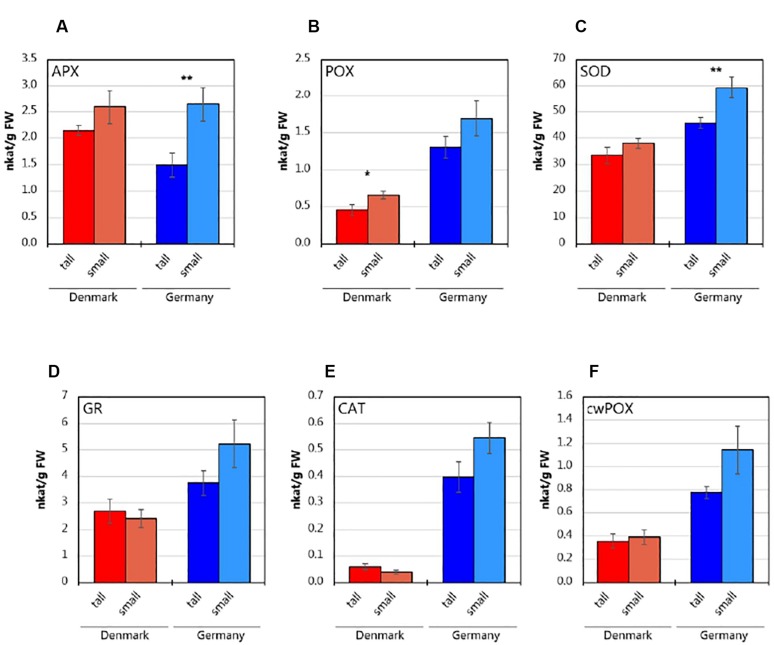
Antioxidative enzyme activities in the roots of tall and small three-year-old *A. nordmanniana*. **(A)** Ascorbate peroxidase (APX). **(B)** Peroxidase (POX). **(C)** Superoxide dismutase (SOD). **(D)** Glutathione reductase (GR). **(E)** Catalase (CAT). **(F)** Cell-wall peroxidase (cwPOX). Values represent mean ± SEM derived from five biological replicates of three technical replicates each. ^∗^, ^∗∗^ indicate statistically significant differences at the *P* < 0.05 and *P* < 0.01 level, respectively, between small and tall plants based on two-sided, unpaired Students *t*-test.

*Abies nordmanniana* plants originating from the German site showed higher activities of the determined antioxidative enzymes in roots (Figure 1), except for APX, which showed similar levels in plants from both sampling sites (Figure 1A). At both sites, root samples from small plants exhibited higher APX activities than the tall plants; the difference was, however, only statistically significant (*P* < 0.01) for the plants from the German site. Similarly, POX (Figure 1B) and SOD (Figure 1C) activities were higher in roots of small plants at both sites, with statistically significant differences (*P* < 0.05) for POX in plants from the Danish site, and for SOD in plants derived from the German site, respectively. The activities of GR (Figure 1D), CAT (Figure 1E) and cwPOX (Figure 1F) were higher by trend in the roots from small plants in Germany. In contrast, roots of the plants from the Danish site did not show consistent differences in these enzyme activities. Overall, the roots from small plants at the German site showed generally higher activity levels of antioxidative enzymes than roots from tall plants, while the differences between tall and small plant enzyme activities were lower at the Danish site.

### Alpha Diversity of Bacterial and Fungal Communities

For bacterial diversity analysis, a total of 3,917,647 16S rRNA gene sequence reads were obtained, and after quality filtering and chimera removal, a total of 1,631,616 sequence reads were used for the analyses. The sequence reads clustered into 28,275 OTUs at 97% sequence similarity level. The rarefaction curves indicated that the number of OTUs increased with the number of sequences read; hence, a deeper sequencing effort is needed to cover the complete diversity of the bacterial communities in the current environments (Supplementary Figure S1). When comparing the rhizosphere bacterial communities from the two sampling sites, a significantly (*P* < 0.05) higher evenness was found for the German versus the Danish rhizosphere samples, while the richness, diversity and evenness indices did not differ between plant sizes (Table 1).

**TABLE 1:** Alpha diversity indices calculated from 16S rRNA sequence data at the operational taxonomic unit (OTU) level of the bacterial communities from rhizosphere of tall and small plants of *A. nordmanniana* collected in Germany and Denmark.

**Diversity indices**				

**Sampling site**	**Plant size**	**Chao 1**	**Shannon**	**Evenness**
Germany	Tall and small	8653	6.636	0.095^∗^
Denmark	Tall and small	8724	6.049	0.061
Germany	Small	9668	6.653	0.086
Germany	Tall	7639	6.619	0.104
Denmark	Small	9058	5.992	0.054
Denmark	Tall	8545	6.122	0.068

*^∗^*t*-test significance codes: 0.05.*

After sequencing of the 18S rRNA gene region, a total of 62,078 paired-end reads were obtained, of which 58,653 could be de-multiplexed using sabre according to the internal barcode tags, while 3,425 pairs of FastQ records did not match the barcodes. The individually quality-filtered datasets using SCATA resulted in a range of 40–60% accepted reads per sample (average = 54%), with a total of 31,820 reads passing QC. The cluster analysis resulted in 516 OTUs (total reads = 22,864) and 8,956 singletons. The rarefaction curves showed that the sequencing effort was not deep enough to cover the complete diversity of fungal communities in the environments under study (Supplementary Figure S2).

A comparison of the richness, diversity and evenness indices for fungal rhizosphere communities between the sampling sites, showed no significant differences. However, a significantly higher (*P* < 0.05) fungal community richness (Chao-1) was found for the small plants at the German site, and significantly higher (*P* < 0.05) fungal community diversity (Shannon) and evenness were found for tall plants at the Danish site (Table 2).

**TABLE 2:** Alpha diversity indices calculated from 18S rRNA sequence data at the OTU level of the fungal of *A. nordmanniana* collected in Germany and Denmark.

**Diversity indices**				

**Sampling site**	**Plant size**	**Chao 1**	**Shannon**	**Evenness**
Germany	Tall and small	350.6	3.185	0.113
Denmark	Tall and small	289.6	2.651	0.090
Germany	Small	373.1^∗^	3.512	0.141
Germany	Tall	328.0	2.858	0.084
Denmark	Small	268.5	2.377	0.063
Denmark	Tall	310.7	2.925^∗^	0.117^∗^

*^∗^*t*-test significance codes: 0.05.*

### Variation in the Structure of *A. nordmanniana* Rhizosphere Microbial Communities

**FIGURE 2:**
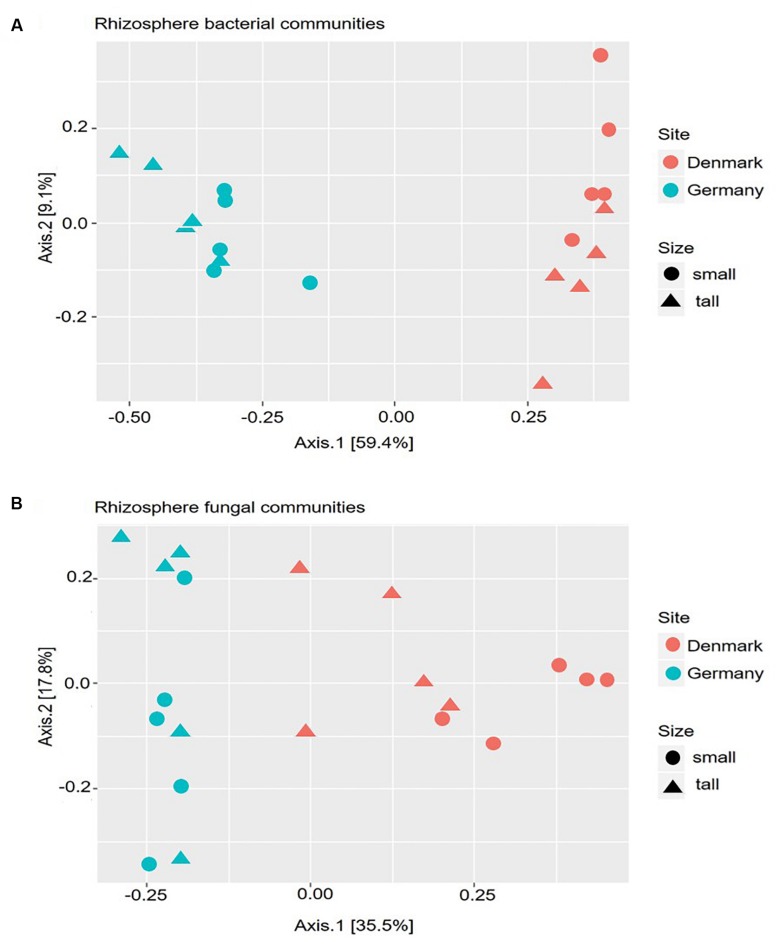
Principal Coordinate Analysis (PCoA) based on the Bray–Curtis dissimilarity matrix between samples. Calculations are based on the operational taxonomic unit (OTU) distribution obtained from amplicon sequencing of the 16S rRNA gene **(A)**, and 18S rRNA gene **(B)**. DNA samples were obtained from rhizosphere of tall and small *A. nordmanniana* plants collected at the German and Danish sampling sites.

The differences in microbial community compositions between rhizosphere samples were measured using a PCoA, based on the relative abundance of OTUs. The PCoA analysis indicated that both bacterial and fungal communities clustered according to the sampling site (Figures 2A,B). Hence, the microbial rhizosphere communities from Germany and Denmark were dissimilar. The non-parametric multivariate analysis performed using the Adonis test at the OTU level, also revealed significant differences in both bacterial and fungal rhizosphere community composition between the sampling sites (Supplementary Table S5). Neither the bacterial nor the fungal community compositions differed significantly between tall and small plants for any of the two sites according to the dissimilarity analysis (Supplementary Table S5).

### Bacterial Community Composition

Across both sampling sites, an overall of 24 bacterial phyla were identified. Proteobacteria was significantly more abundant than any other phylum (*P* < 0.05), with relative abundances of 27–50%, but the relative abundance was not significantly different between sampling sites. Actinobacteria, Acidobacteria as well as Bacteriodetes were also abundant with relative abundances above 20% (Supplementary Figure S3). At sampling site level, the relative abundance of Actinobacteria was significantly higher (*P* < 0.05) in the Danish samples, while the relative abundance of Bacteriodetes, Firmicutes, and Verrucomicrobia were significantly higher (*P* < 0.05) in the German samples.

**FIGURE 3:**
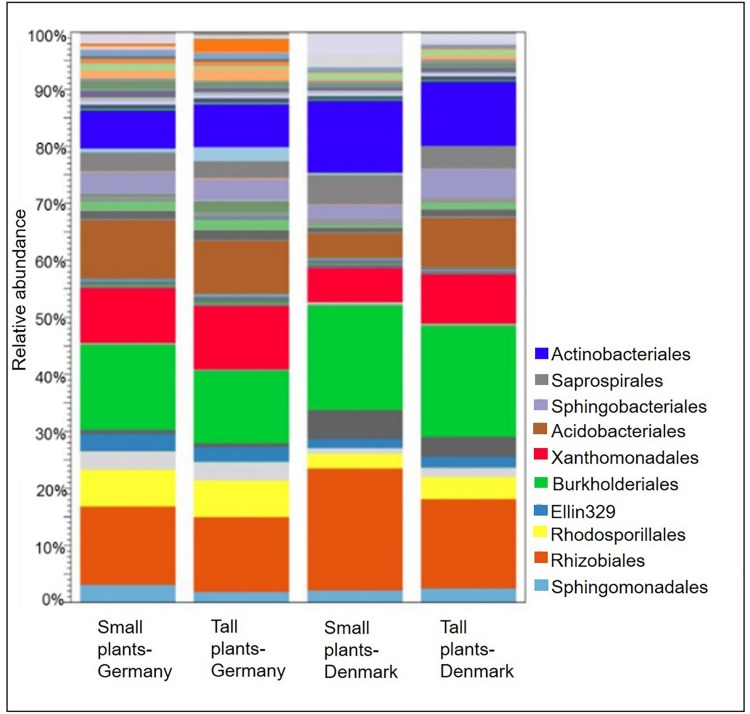
Stacked bar plots showing relative abundances of bacterial orders in the rhizosphere of tall and small *A. nordmanniana* from German and Danish sampling sites. Plots are based on sequencing data for 16S rRNA genes.

At the bacterial order level, the relative abundances of Actinomycetales, Burkholderiales, and Rhizobiales, were higher in the rhizosphere of plants collected in Denmark as compared to Germany (*P* < 0.05). Nevertheless, they constituted the three most abundant orders among all samples. Furthermore, the relative abundances of Acidobacteriales, Rhodospirillales and Xanthomonadales, were significantly (*P* < 0.05) higher in rhizosphere from plants collected in Germany (Figure 3). A SIMPER analysis identified Acidobacteriales, Actinomycetales, Burkholderiales, Rhizobiales, and Xanthomonadales as the taxa that accounted for 46% of the dissimilarities between the Danish and German samples (Supplementary Table S6).

At the plant size level, Acidobacteriales and Xanthomonadales were particularly more abundant (*P* < 0.05) in the rhizosphere from tall plants in Danish samples, compared with the corresponding small plants. Additionally, the relative abundances of Burkholderiales and Rhizobiales were higher in the small plants from both sampling sites, however, the differences were not statistically significant.

**FIGURE 4:**
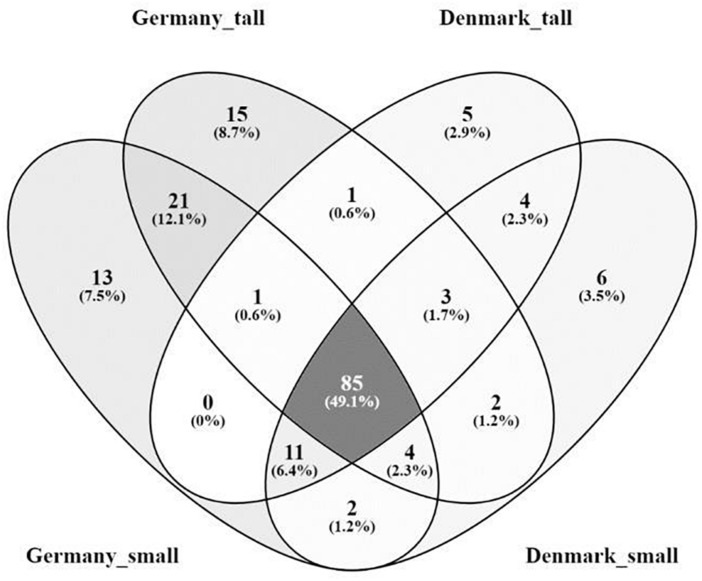
Venn diagram of the rhizosphere core bacterial microbiome from tall and small plants of *A. nordmanniana* collected in Germany and Denmark. Analysis was carried out at the order level. Numbers represent the amount of shared and unique OTUs for each variable. The numbers in parentheses show the percentage of contribution of the core OTUs in relation to the total number of OTUs identified.

The *A. nordmanniana* bacterial core microbiome constituted 49.1% of the identified OTUs at the order level, which were shared across the two sampling sites and across plant sizes (Figure 4); it was dominated by OTUs representing the Acidobacteriales, Actinomycetales, Burkholderiales, Rhizobiales, and Xanthomonadales (Supplementary Figure S4). The German samples presented a higher percentage of unique OTUs 28.3% compared with the Danish samples (8.7%, Figure 4). For the OTUs exclusively found in the microbiomes of small plants there was little overlap between German and Danish samples and the same was the case for OTUs exclusively found in tall plant microbiomes (Figure 4 and Supplementary Figure S5); overall, these unique taxa presented low relative abundances.

**FIGURE 5:**
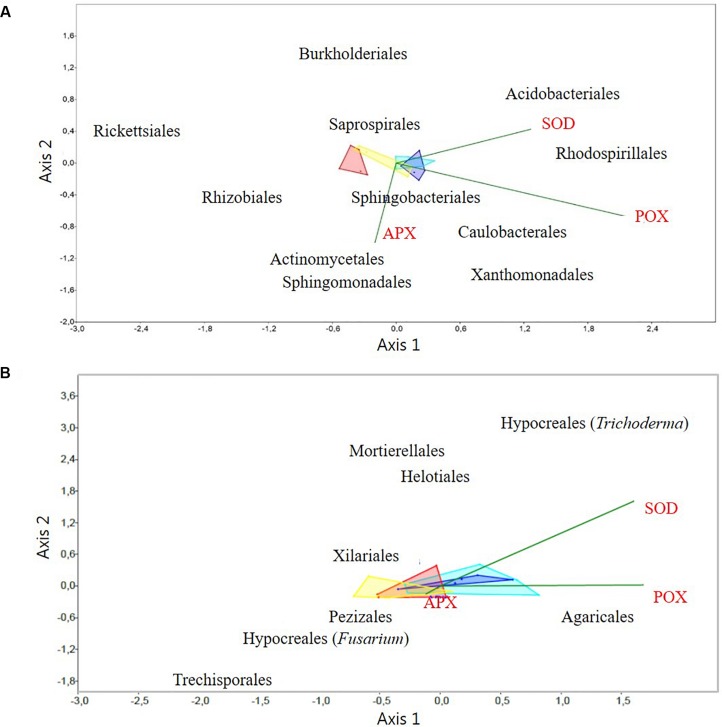
Canonical correspondence analysis (CCA) of activities of antioxidative plant enzymes versus: **(A)** the relative abundance of rhizosphere bacterial OTUs at the order level, and **(B)** the relative abundance of rhizosphere fungal OTUs at the order level. Only the discriminatory taxa (according to SIMPER analysis), and enzyme results with significant differences (*P* < 0.05): Superoxide dismutase (SOD), Ascorbate peroxidase (APX), and Peroxidase (POX), were included in the CCA analyses. Light and dark blue areas correspond to samples from Germany, from small and tall plants, respectively. Red and yellow correspond to samples from Denmark, from small and tall, plants, respectively. The dark green line vectors represent the direction of the root antioxidant enzymes levels and the bacterial and fungal OTUs correlations.

Correlations between activities of antioxidative enzymes and the relative abundances of the taxa at the order level, which contributed at least 3% to the variation between samples according to SIMPER (Supplementary Table S6), were determined by a CCA. The analyses showed correlations for several taxa to one or more enzyme activity. Hence, relative abundances of Actinomycetales, Rhizobiales, Sphingobacteriales, and Xanthomonadales were positively correlated with APX (*P* < 0.05); abundances of Acidobacteriales, Burkholderiales, Rhodospirillales, Saprospirales and Xanthomonadales were positively correlated with SOD (*P* < 0.05); and Acidobacteriales, Caulobacterales, Saprospirales, and Sphingomonadales were positively correlated with POX (*P* < 0.001). In contrast only Burkholderiales was negatively correlated with APX (*P* < 0.01) and Actinomycetales and Rhizobiales with SOD (*P* < 0.05). Finally, Rickettsiales did not show any significant correlation with the analyzed enzymes (Figure 5A).

### Fungal Community Composition

The 18S rRNA amplicon-targeted analysis identified three fungal phyla, Ascomycota, Basidiomycota and Murocomycota. OTUs that could be assigned to oomycetes, plasmodiophorids or nematodes represented less than 3% the total DNA amplified and were consequently left out of further analyses.

Overall, Ascomycota was more abundant in the rhizosphere of plants collected from Denmark, with a relative abundance of 75–90%, while in German samples, Ascomycota showed a relative abundance of 45–60%, but these differences were not statistically significant. Additionally, for the German samples, Basidiomycota was found in significantly (*P* < 0.05) higher relative abundance (30–50%) compared with the Danish samples (10–20%; Supplementary Figure S6). The relative abundance of Mucoromycota was also higher compared with the samples from Denmark but these differences were not statistically significant; nevertheless, this phylum had the lowest overall relative abundance at both sampling sites. When comparing the relative abundances of fungal phyla at plant size level, Ascomycota dominated in tall and small plants from Denmark and in the small plants from Germany. For the tall plants in Germany, Basidiomycota was the dominating phylum; however, this dominance was not significant (Supplementary Figure S6).

**FIGURE 6:**
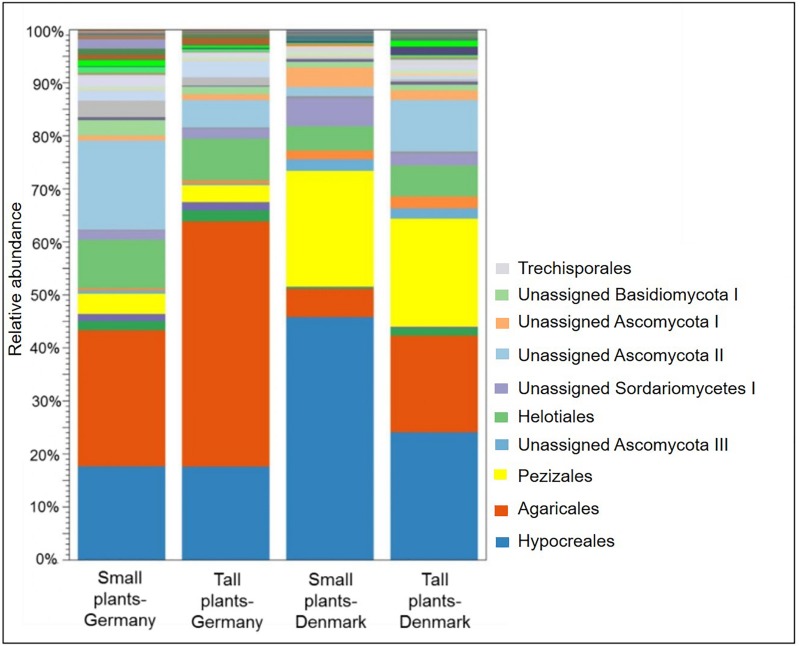
Stacked bar plot showing relative abundances of fungal order in the rhizosphere of tall and small *A. nordmanniana* plants from German and Danish sampling sites. Plots are based on sequencing data for 18S rRNA genes.

A comparison of fungal community composition at the order level between the sites showed that Agaricales was significantly (*P* < 0.05) more abundant in rhizosphere samples from Germany, while Hypocreales and Pezizales was significantly (both *P* < 0.05) more abundant in the Danish rhizosphere samples (Figure 6). SIMPER analysis identified Agaricales, Hypocreales and Pezizales as the taxa that each accounted for at least 20% of the dissimilarities between the Danish and German samples (Supplementary Table S7). Further, the order Hypocreales was dominated by the genus *Fusarium*. Hence, according to SIMPER analysis, *Fusarium* was the genus that contributed the most (21%) to the dissimilarity of fungal communities between the sampling sites (Supplementary Table S8). Plant size had a significant effect on the relative abundance of Agaricales (*P* < 0.05), which was higher in the tall plants when comparing with the small plants at both sampling sites. Contrary the order Hypocreales had a significantly (*P* < 0.05) higher relative abundance in the Danish small plants than in Danish tall plants (Figure 6).

**FIGURE 7:**
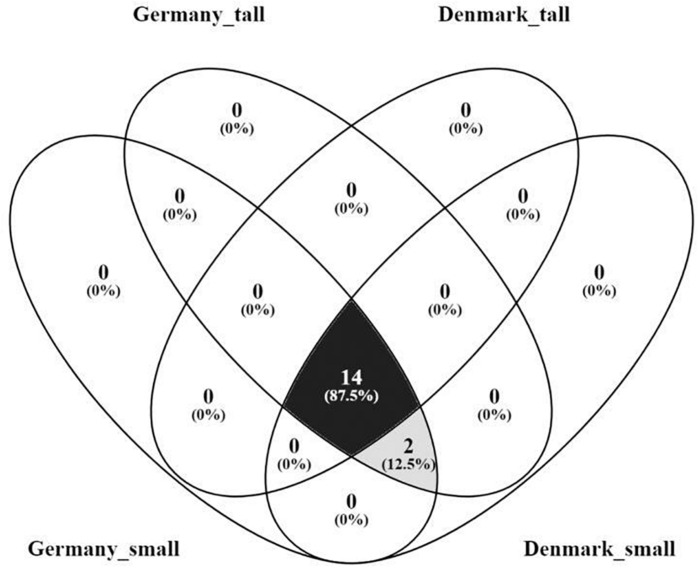
Venn diagram of the core rhizosphere fungal microbiome of *A. nordmanniana* from tall and small plants collected in Germany and Denmark. Analysis was carried out at the order level. Numbers represent the amount of shared and unique OTUs for each variable. The numbers in parentheses show the percentage of contribution of the core OTUs in relation to the total number of OTUs identified.

The core fungal microbiome constituted 87.5% of the OTUs identified (order level) (Figure 7) were shared across the sampling sites and plant sizes and was dominated by Agaricales, Hypocreales and Pezizales (Supplementary Figures S7A,B). We did not find OTUs that were unique for small or tall plant microbiomes (Supplementary Figure S7B).

A search for correlations between the relative abundance of fungi at the order level (within the Hypocreales; two genera *Trichoderma* and *Fusarium* could be analyzed independently), and activities of antioxidative enzymes by CCA, revealed that the relative abundance of *Fusarium* (Hypocreales) was significantly (*P* < 0.01) negatively correlated to APX and SOD. The relative abundance of *Agaricales* was significantly (*P* < 0.05) positively correlated to POX. Finally, the relative abundance of the genus *Trichoderma* (Hypocreales) was significantly (*P* < 0.01) positively correlated to SOD. The fungal orders Helotiales, Mortierellales, Trechisporales, Pezizales and Xilariales, did not show any significant correlations to the analyzed antioxidative enzymes (Figure 5B).

## Discussion

The current study of the bacterial and fungal communities naturally associated with the rhizosphere of *A. nordmanniana* is, to our knowledge, the first study documenting the root-associated microbial diversity in relation to plant growth for this Christmas tree species. The most abundant rhizosphere bacteria phyla at both sampling sites (Acidobacteria, Actinobacteria, Bacteriodetes, and Proteobacteria), have been reported before as dominating bacterial communities associated with conifers (Yu et al., 2013; Naumova et al., 2015; Shi et al., 2015; Proença et al., 2016; Uroz et al., 2016). For the fungal communities, Ascomycota and Basidiomycota were the most abundant and common fungal phyla. Our findings are in agreement with previous studies that reported Ascomycota to be associated with conifers (Yu et al., 2013; Stenstrom et al., 2014), while Basidiomycota appears to be the major fungal phylum in the *Pinus tebulaeformis* rhizosphere (Yu et al., 2013).

### Root-Associated Core Microbiome of *A. nordmanniana*

The *A. nordmanniana* fungal core microbiome comprised taxa within the orders of Agaricales (Basidiomycota), Hypocreales (Ascomycota) and Pezizales (Ascomycota). Ascomycetes might have roles in nutrient acquisition and transformation of organic nitrogen and phosphorus into their inorganic forms (Stenstrom et al., 2014), and therefore may contribute significantly to the nutrient supply needed for maintaining plant growth, but the order Hypocreales even contains important plant pathogens (Dean et al., 2012). Members of the order of Pezizales might both have biotrophic and decomposing potential (Vrålstad et al., 1998). Finally, the high abundance of Agaricales, which comprises many EM species (Tedersoo and Smith, 2013), points toward ectomycorrhization of *A. nordmanninana* in the studied field sites, and ectomycorrhization in *Abies* spp. has been previously reported (Taylor et al., 2000; Rudawska et al., 2016; Oros-Ortega et al., 2017). However, in our study, and for Agaricales, it was not possible to assign OTUs to lower taxa to confirm ectomycorrhization of our root samples.

The bacterial core microbiome of *A. nordmanniana* was dominated by the orders Burkholderiales and Rhizobiales, but even included Acidobacteriales, Actinomycetales and Xanthomonadales. Non-nodulating *Rhizobium* spp. and some members of Burkholderiales, as well as of Xanthomonadales, and Actinomycetales are dominantly detected on the ectomycorrhizal (EM) root tips of many conifer species (Khetmalas et al., 2002; Izumi et al., 2006; Burke et al., 2008; Nguyen and Bruns, 2015; Lladó et al., 2017). Therefore, the high abundance of these taxa associated with roots of *A. nordmanniana* could be influenced by their interactions with EM (Nguyen and Bruns, 2015). Additionally, Burkholderiales and Rhizobiales, includes nitrogen-fixing and mineral-weathering bacteria (Suna et al., 2014) and are part of the most abundant root-associated bacterial orders and core microbiomes for a wide range of plant hosts (Yeoh et al., 2017; Garrido-Oter et al., 2018).

Root exudates have been reported to play an important role in shaping the root-associated microbial communities (Chaparro et al., 2013). Hence, the ability of *A. nordmanniana* to maintain a core microbiome may be ascribed to specific nutrients and signals in these exudates. Moreover, the possible intraspecific plant genetic variation can alter root-associated microbial communities, thus the interaction of the plant genotype and the soil environment might contribute to the root-microbiome assembly and their complexity in natural environments (Lundberg et al., 2012; Wagner et al., 2016). Furthermore, even different soil characteristics and other factors like climate influence the rhizosphere microbiome associated with a particular plant species (Costa et al., 2006). For the current study including two sandy soils, we observed different relative abundances of microbial taxa between the two sites, as well as a site effect on the bacterial species evenness. These results support the notion that the soil type is an important factor shaping the composition of root-associated microbial communities as seen for other plant species (Mendes et al., 2013; Schreiter et al., 2014; Vanderkoornhuyse et al., 2015). Furthermore, the two sampling sites presented differences in the rainfall during the period prior to sampling. This could have an influence on the microbial communities associated with the sampled rhizospheres, as soil moisture is a key environmental component shaping soil and rhizosphere microbial communities. For example, periods of drought and rewetting could alter the microbial community structure (Cruz-Martínez et al., 2012; Barnard et al., 2013; Chodak et al., 2014).

### Root-Associated Microbiome Versus *A. nordmanniana* Growth Phenotype

We analyzed the composition of rhizosphere microbial communities for small (growth-retarded) versus tall plants to look for biotic causes or consequences of the growth retardation. The microbiome of small, growth-retarded plants was generally characterized by a higher relative abundance of *Fusarium* spp., and a lower relative abundance of *Agaricales* compared to tall plants. The dissimilarities are even reflected in significant differences at plant size level in the fungal community richness, diversity and evenness. *Fusarium* is a diverse genus including plant pathogenic species (Dean et al., 2012), and many conifers such as *Abies* spp. are affected by *Fusarium* infections (Gordon et al., 2015), while some *Fusarium* species have been described as potential beneficial endophytes, with insecticide and nematocide properties (Vu et al., 2006; Paparu et al., 2008). Associations of *Fusarium* spp. with roots of conifers have been reported before (Lilja et al., 1997; Stenstrom et al., 2014). A generally higher abundance of *Fusarium* spp. has previously been found in the rhizosphere of diseased clonal *Picea mariana* plants compared to healthy plants (Vujanovic et al., 2007), and these authors indicated that plant health was influenced by the composition of taxa among diseased and healthy *P. mariana*. The plants we sampled in the current study seemed healthy despite their size difference. However, the higher abundance of *Fusarium* in the small plants might indicate that some of these fungi could represent latent pathogens, with a potential to cause stress in the small plants. Nevertheless, the identified *Fusarium* OTUs could also represent part of the fungal biota in the root-attached soil, and not necessary represent latent pathogens inside the sampled roots. Therefore, future studies aiming to a deeper understanding of the interactions between *Fusarium* spp. and *A. nordmanniana*, should distinguish the fungal microbiota associated to root tissues, from the one in the rhizosphere soil. Additionally, such studies should employ sequence analysis targeting the ITS region and include a cultivation approach to obtain fungal isolates for detailed investigations.

Alternatively, the order Agaricales that showed a higher relative abundance in tall plants, contains several potential beneficial plant-growth promoting and EM-forming species (Tedersoo and Smith, 2013; Contreras-Cornejo et al., 2015) that may counteract biotic and abiotic stressors. Hence, the tall plants may experience less stress or better nutrient uptake than the small plants, due to beneficial effects by Agaricales spp. (Contreras-Cornejo et al., 2015; Floudas et al., 2015; Rudawska et al., 2016), thus allowing for more resources being allocated to growth. It has been suggested that some fungal species associated with healthy plants could have the potential to compete against root pathogens in the rhizosphere of *Picea* spp., thus minimizing their pathogenic effect or/and reducing their abundance in healthy plants (Vujanovic et al., 2007).

Hence, an increased relative abundance of potential latent pathogens included in *Fusarium* spp., as well as a decreased relative abundance of potentially EM forming members of Agaricales, could be important for the observed plant size differences. Further, the relative abundance of the bacterial order Xanthomonadales was lower in small, growth-retarded plants. Xanthomonadales contain taxa that can cause diseases in conifers, but are even associated with EM (Khetmalas et al., 2002). Their lower abundance in small plants, may be related to the low abundance of Agaricales. We suggest that bacterial disease could not be the major driver for plant size differences.

### Root Antioxidative Enzyme Activity Profiles of *A. nordmanniana*

Results on antioxidative enzyme activities may contribute to the understanding of the plant growth phenotype, due to their essential involvement in regulating growth-related processes and stress responses (Apel and Hirt, 2004; Das et al., 2015). It has previously been reported that an increase in the antioxidative enzyme levels in plants occurs as a mechanism to reduce oxidative stress caused by biotic or abiotic factors (Bharti et al., 2016; Caverzan et al., 2016; Sarkar et al., 2018), and that plants lower their growth in response to stress (Zhu, 2001).

Interestingly, the plants in Germany showed higher antioxidative enzyme activities than plants at the Danish site. Activity profiles of antioxidative enzymes integrate the plant response to abiotic and/or biotic stressors experienced over a longer time than transcriptome profiles (Rasmussen et al., 2013). Hence, the different antioxidative enzymes levels for plants from the two sampling sites, points to differential exposure to biotic and/or abiotic factors prior harvesting. Considering the highly variable precipitation recorded for the German sampling site, we propose that transient exposure to drought, which increases ROS levels in plants (Mittler, 2002), could contribute to the high antioxidative enzyme activities. Moreover, the generally higher enzyme activities observed in small plants, particularly at the German site, could be indicative for increased ROS levels. Subsequently, an increase in ROS levels may demand more plant resources, to cope with the stress and thus limiting plant growth (Zhu, 2001; Caverzan et al., 2016). With the current approach, however, the specific stress source(s) and detailed mechanisms whereby the plants cope with the stress and/or regulate growth, differentially in the tall and small plants could not be resolved.

### *Abies nordmanniana* Root-Associated Microbiome Correlation With Root Antioxidative Enzyme Activity Profiles

We observed that many more bacterial taxa correlated positively than negatively with one or more antioxidative enzyme activity. The ability of bacteria to impact plant antioxidative enzyme activities has primarily been determined for introduced plant beneficial strains and primarily under controlled conditions, where salt or water stress could be imposed on the plants (Jebara et al., 2005; Bharti et al., 2016; Vurukonda et al., 2016). Under such conditions, the introduced bacteria may either increase (Helepciuc et al., 2014; Bharti et al., 2016; Chandra et al., 2018) or decrease the enzyme activities (Vardharajula et al., 2011). The different responses are probably caused by the different mechanisms whereby bacteria can affect plant stress physiology. Some bacteria can increase plant antioxidative enzyme activities directly by interfering with plant stress signaling (Jebara et al., 2005; Vurukonda et al., 2016), while others decrease the activities due to their ability to ameliorate stress, as seen for drought stress by exopolysaccharide production (Vardharajula et al., 2011).

For the fungal taxa, *Fusarium* correlated negatively with APX and SOD, while Agaricales correlated positively to POX. In contrast to the current finding, an increase in POX activity has been observed at the initial stages of the root necrosis infection with *Fusarium* spp. in *Picea abies* and *Pinus silvestris* (Asiegbu et al., 1999). Studies for other plant species like chickpea, have even documented an increase in antioxidative enzymes during confrontation with *Fusarium*, as well as a time-dependent enzymatic response reliant on compatibility between host and pathogen (Garcia-Limones et al., 2009; Dalvi et al., 2017). The relation between Agaricales and antioxidative enzyme activities has not previously been addressed for *Abies* or other conifers. However, our results suggest that the establishment of associations with plant beneficial members of this fungal group, such as EM species (Tedersoo and Smith, 2013), might affect these enzyme activities. For comparison, arbuscular mycorrhizal fungi can both, decrease and increase antioxidative enzyme activities e.g., under drought stress (Armada et al., 2016).

In summary, the current data show a relationship between the composition of rhizosphere microbiota, the plant antioxidative enzymes, and the plant growth habit. They indicate that the microbiota have a strong influence on the cellular metabolism in the roots, and that their ability to increase plant antioxidative enzyme defenses is widespread, or that the plant root recruits beneficial microbes under stressful conditions. Nevertheless, from this field study, we cannot disentangle the causes from the consequences regarding these correlations. However, we suggest that future studies may provide novel knowledge about the possible mechanisms that different microbial groups employ during association with stressed plants. Finally, many of the bacterial and fungal taxa that differ in relative abundance between sites or between tall and small are important for nutrient turnover in soil and rhizosphere. Hence, future studies should focus on relations between the relative abundance of these taxa, soil or plant nutrient concentrations, and the activities of enzymes involved in soil nutrient transformations.

## Data Availability

The datasets generated for this study can be found in the PRJNA515250.

## Author Contributions

AG-L contributed to the experimental design, the sampling, the acquisition and analysis of the data and the interpretation, and the writing of the manuscript. DG contributed to the data acquisition and analysis of plant antioxidative enzymes, and the writing of the manuscript. MS contributed to the eukaryotic gene amplification and amplicon sequencing, the processing of 18S rDNA sequencing data, and the writing of the manuscript. OL contributed to the processing and interpretation of 18S rDNA sequencing data and the revising of the manuscript. MN contributed to experimental design and data analysis, and the revising of the manuscript. TR contributed to the data analysis, and the revising of the manuscript. BV contributed to the experimental design, the sampling, and the revising of the manuscript. ON contributed to the experimental design, the sampling, the analysis of the data and interpretation, and the writing of the manuscript.

## Conflict of Interest Statement

The authors declare that the research was conducted in the absence of any commercial or financial relationships that could be construed as a potential conflict of interest.

## Footnotes

1 http://www.dwd.de

2 http://www.dmi.dk

3 https://www.qiagenbioinformatics.com/

4 https://github.com/najoshi/sabre

5 https://scata.mykopat.slu.se/

6 http://www.ncbi.nlm.nih.gov/

7 http://www.arb-silva.de/browser/

## References

[B1] Aebi H. (1984). Catalase *in vitro*. *Methods Enzymol.* 105 121–126. 6727660 10.1016/s0076-6879(84)05016-3

[B2] Apel K. Hirt H. (2004). Reactive oxigen species: metabolism, oxidative stress, and signal transduction. *Annu. Rev. Plant Biol.* 55 373–399. 10.1146/annurev.arplant.55.031903.141701 15377225

[B3] Armada E. Probanza A. Roldán A. Azcóna R. (2016). Native plant growth promoting bacteria Bacillus thuringiensis and mixed or individual mycorrhizal species improved drought tolerance and oxidative metabolism in *Lavandula dentata* plants. *J. Plant Physiol.* 192 1–12. 10.1016/j.jplph.2015.11.007 26796423

[B4] Asiegbu F. O. Kacprzak M. Daniel G. Johansson M. Stenlid J. Mañka M. (1999). Biochemical interactions of conifer seedling roots with *Fusarium* spp. *Can. J. Microbiol.* 45 923–935. 10.1139/w99-099

[B5] Bais H. P. Weir T. L. Perry L. G. Gilroy S. Vivanco J. M. (2006). The role of root exudates in rhizosphere interactions with plants and other organisms. *Annu. Rev. Plant Biol.* 57 233–266. 10.1146/annurev.arplant.57.032905.105159 16669762

[B6] Barnard R. L. Osborne C. A. Firestone M. K. (2013). Responses of soil bacterial and fungal communities to extreme desiccation and rewetting. *ISME J.* 7 2229–2241. 10.1038/ismej.2013.104 23823489 PMC3806258

[B7] Bharti N. Pandey S. Barnawal D. Patel P. Kalra A. (2016). Plant growth promoting rhizobacteria *Dietzia natronolimnaea* modulates the expression of stress responsive genes providing protection of wheat from salinity stress. *Sci. Rep.* 6:34768. 10.1038/srep34768 27708387 PMC5052518

[B8] Bräuner Nielsen U. Hansen J. K. Kromann H. K. (2011). Impact of site and provenance on economic return in Nordmann fir Christmas tree production. *Scand. J. Forest Res.* 262 74–89. 10.1080/02827581.2010.526955

[B9] Brotman Y. Landau U. Cuadros-Inostroza Á Takayuki T. Fernie A. R. Chet I. (2013). *Trichoderma*-plant root colonization: escaping early plant defense responses and activation of the antioxidant machinery for saline stress tolerance. *PLoS Pathog.* 9:e1003221. 10.1371/journal.ppat.1003221 23516362 PMC3597500

[B10] Burke D. J. Dunham S. M. Kretzer A. (2008). Molecular analysis of bacterial communities associated with the roots of *Douglas fir* Pseudotsuga menziesii colonized by different ectomycorrhizal fungi. *FEMS Microbiol. Ecol.* 65 299–309. 10.1111/j.1574-6941.2008.00491.x 18459969

[B11] Caverzan A. Casassola A. Brammer S. P. (2016). Antioxidant responses of wheat plants under stress. *Genet. Mol. Biol.* 39 1–6. 10.1590/1678-4685-GMB-2015-0109 27007891 PMC4807390

[B12] Chandra D. Srivastava R. Glick B. R. Sharma A. K. (2018). Drought- Tolerant *Pseudomonas* spp. improve the growth performance of finger millet (*Eleusine coracana* (L.) gaertn.) under non-stressed and drought-stressed Conditions. *Pedosphere* 28 227–240. 10.1016/S1002-0160(18)60013-X

[B13] Chaparro J. M. Badri D. V. Bakker M. G. Sugiyama A. Manter D. K. Vivanco J. M. (2013). Root Exudation of phytochemicals in *Arabidopsis* follows specific patterns that are developmentally Pprogrammed and correlate with soil microbial functions. *PLoS One* 82:1–10. 10.1371/journal.pone.0055731 23383346 PMC3562227

[B14] Chodak M. Gołębiewski M. Morawska-Płoskonka J. Kuduk K. Niklińska M. (2014). Soil chemical properties affect the reaction of forest soil bacteria to drought and rewetting stress. *Ann. Microbiol.* 65 1627–1637. 10.1007/s13213-014-1002-0 26273241 PMC4529456

[B15] Clarke K. R. Gorley R. N. (2006). *Primer v6: User Manual/Tutorial.* Plymouth: PRIMER-E.

[B16] Clemmensen K. E. Ihrmark K. Brandström-Durling M. Lindahl B. D. (2016). “Sample preparation for fungal community analysis by high-throughput sequencing of barcode amplicons,” in *Microbial Environmental Genomics MEG*, eds Martin F. Uroz S. (New York, NY: Springer), 61–88. 10.1007/978-1-4939-3369-3_4

[B17] Contreras-Cornejo H. A. Macías-Rodríguez L. López-Bucio J. (2015). “Fungal biomolecules in plant growth promotion,” in *Fungal Biomolecules*, eds Gupta V. K. Mach R. L. Sreenivasaprasad S. (Hoboken, NJ: WILEY), 10.1002/9781118958308.ch23

[B18] Costa R. Gotz M. Mrotzek N. Lottmann J. Berg G. Smalla K. (2006). Effects of site and plant species on rhizosphere community structure as revealed by molecular analysis of microbial guilds. *FEMS Microbiol. Ecol.* 56 236–249. 10.1111/j.1574-6941.2005.00026 16629753

[B19] Cruz-Martínez K. Rosling A. Zhang Y. Song M. Andersen G. L. Banfield J. F. (2012). Effect of rainfall-induced soil geochemistry dynamics on grassland soil microbial communities. *Appl. Environ. Microbiol.* 78 7587–7595. 10.1128/AEM.00203-12 22904056 PMC3485702

[B20] Dalvi U. Naik R. Kale A. (2017). Antioxidative enzyme responses against Fusarium wilt *Fusarium oxysporum f. sp. ciceri*s in chickpea genotypes. *Annu. Res. Rev. Biol.* 12 1–9. 10.9734/ARRB/2017/32888

[B21] Das P. Nutan K. K. Singla-Pareek S. L. Pareek A. (2015). Oxidative environment and redox homeostasis in plants: dissecting out signifcant contribution of major cellular organelles. *Front. Environ. Sci.* 2:70 10.3389/fenvs.2014.00070

[B22] Dean R. Van Kan J. A. L. Pretorius Z. A. Hammond-Kosack K. E. Di Pietro A. Spanu P. D. (2012). The Top 10 fungal pathogens in molecular plant pathology. *Mol. Plant Patholol.* 13 414–430. 10.1111/j.1364-3703.2011.00783.x 22471698 PMC6638784

[B23] Desjardins P. Conklin D. (2010). NanoDrop microvolume quantitation of nucleic acids. *J. Vis. Exp.* 45:e2565. 10.3791/2565 21189466 PMC3346308

[B24] Edwards E. A. Rawsthorne S. Mullineaux P. M. (1990). Subcellular distribution of multiple forms of glutathione reductase in leaves of pea *Pisum sativum* L. *Planta* 180 278–284. 10.1007/BF00194008 24201957

[B25] Fadrosh D. W. Ma B. Gajer P. Sengamalay N. Ott S. Brotman R. M. (2014). An improved dual-indexing approach for multiplexed 16S rRNA gene sequencing on the Illumina MiSeq platform. *Microbiome* 2:6. 10.1186/2049-2618-2-6 24558975 PMC3940169

[B26] Floudas D. Held B. W. Riley R. Nagy G. L. Koehler G. Ransdell A. (2015). Evolution of novel wood decay mechanisms in Agaricales revealed by the genome sequences of *Fistulina hepatica* and *Cylindrobasidium torrendii*. *Fungal Genet. Biol.* 76 78–92. 10.1016/j.fgb.2015.02.002 25683379 PMC4399860

[B27] Garcia-Limones C. Dorado G. Navas-Cortés J. A. Jiménez-Díaz R. M. Tena M. (2009). Changes in the redox status of chickpea roots in response to infection by *Fusarium oxysporum* f. *sp. ciceris*: apoplastic antioxidant enzyme activities and expression of oxidative stress-related genes. *Plant Biol.* 11 194–203. 10.1111/j.1438-8677.2008.00095.x 19228326

[B28] Garrido-Oter R. Nakano R. T. Dombrowski N. Ma K. W. AgBiome Team, McHardy A. C. (2018). Modular traits of the Rrhizobiales root microbiota and their evolutionary relationship with symbiotic rhiozobia. *Cell Host Microbe* 24 155–167. 10.1016/j.chom.2018.06.006 30001518 PMC6053594

[B29] Gill S. S. Tuteja N. (2010). Reactive oxygen species and antioxidant machinery in abiotic stress tolerance in crop plants. *Plant Physiol. Biochem.* 48 909–930. 10.1016/j.plaphy.2010.08.016 20870416

[B30] Gordon T. R. Swett C. L. Wingfield M. (2015). Management of *Fusarium* diseases affecting conifers. *Crop Protect.* 73 28–39. 10.1016/j.cropro.2015.02.018

[B31] Hanlon E. A. (2012). Soil pH and Electrical Conductivity: A County Extension Soil Laboratory Manual. Cir1081. UF/IFAS Extension. Available at: http://edis.ifas.ufl.edu/ss118 (accessed July 9, 2016).

[B32] Hardoim P. R. van Overbeek L. S. Berg G. Pirttilä A. M. Compant S. Sessitsch A. (2015). The hidden world within Plants: ecological and evolutionary considerations for defining functioning of microbial endophytes. *Microbiol. Mol. Biol. Rev.* 793 293–320. 10.1128/MMBR.00050-14 26136581 PMC4488371

[B33] Helepciuc F. E. Mitoi M. E. Manole-Paunescu F. A. Breseanu A. Cornea C. P. (2014). Induction of plant antioxidant system by interaction with beneficial and/or pathogenics microorganisms. *Roman. Biotechnol. Lett.* 19 9366–9375.

[B34] Herlemann D. P. R. Labrenz M. Jürgens K. Bertilsson S. Waniek J. J. Andersson A. F. (2011). Transitions in bacterial communities along the 2000 km salinity gradient of the Baltic Sea. *ISME J.* 5 1571–1579. 10.1038/ismej.2011.41 21472016 PMC3176514

[B35] Izumi H. Anderson I. C. Alexander I. J. Killham K. Moore E. R. B. (2006). Endobacteria in some ectomycorrhiza of Scots pine *Pinus sylvestris*. *FEMS Microbiol. Ecol.* 56 34–43. 10.1111/j.1574-6941.2005.00048.x 16542403

[B36] Jammer A. Gasperl A. Luschin-Ebengreuth N. Heyneke E. Chu H. Cantero-Navarro E. (2015). Simple and robust determination of the activity signature of key carbohydrate metabolism enzymes for physiological phenotyping in model and crop plants. *J. Exp. Bot.* 66 5531–5542. 10.1093/jxb/erv228 26002973

[B37] Jebara S. Jebara M. Limam F. Aouani M. (2005). Changes in ascorbate peroxidase, catalase, guaiacol peroxidase and superoxide dismutaseactivities in common bean nodules under salt stress. *J. Plant Physiol.* 162 929–936. 10.1016/j.jplph.2004.10.005 16146319

[B38] Khetmalas M. B. Egger K. N. Massicotte H. B. Tackaberry L. E. Clapperton M. J. (2002). Bacterial diversity with subalpine fir *Abies lasiocarpa* ectomycorrhizae following wildfire and salvage-logging in central British Columbia. *Can. J. Microbiol.* 48 611–662. 12224560 10.1139/w02-056

[B39] Kohler J. Hernandez J. Caravaca F. C. Roldan A. (2008). Plant-growth-promoting rhizobacteria and arbuscular mycorrhizal fungi modify alleviation biochemical mechanisms in water-stressed plants. *Funct. Plant Biol.* 35 141–151. 10.1071/FP07218 32688765

[B40] LaFever R. E. Vogel B. S. Croteau R. (1994). Diterpenoid resin acid biosynthesis in Conifers: Enzymatic cyclization of geranylgeranyl pyrophosphate to abietadiene, the precursor of abietic acid. *Arch. Biochem. Biophys.* 3131 139–149. 10.1006/abbi.1994.1370 8053674

[B41] Lilja A. Lilja S. Kurkela T. Rikala R. (1997). Nursery practices and management of fungal diseases in forest nurseries in Finland. A review. *Silva Fennica* 311 79–100.

[B42] Lladó S. Lopez-Mondejar R. Baldrian P. (2017). Forest soil bacteria: diversity, involment in ecosystem processes, and response to global change. *Microbiol. Mol. Biol. Rev.* 81 e63–16. 10.1128/MMBR.00063-16 28404790 PMC5485800

[B43] Lundberg D. S. Lebeis S. L. Paredes S. H. Yourstone S. Gehring J. Malfatti S. (2012). Defining the core *Arabidopsis thaliana* root microbiome. *Nature* 488 86–90. 10.1038/nature11237 22859206 PMC4074413

[B44] Mandal S. Mitra A. Mallick N. (2008). Biochemical characterization of oxidative burst during interaction between *Solanum lycopersicum* and *Fusarium oxysporum f. sp. lycopersici*. *Physiol. Mol. Plant Pathol.* 72 56–61. 10.1016/j.pmpp.2008.04.002

[B45] Marx D. H. (1970). The Influence of ectotrophic mycorrhizal fungi on the resistance of pine roots to pathogenic infections. V. Resistance of mycorrhizae to infection by vegetative mycelium of *Phytophthora cinnamomi*. *phytopathology* 60 1472–1473. 10.1094/Phyto-60-1472 5811914

[B46] McCord J. M. Fridovich I. (1969). Superoxide dismutase: an enzymic function for erythrocuprein hemocuprein. *J. Biol. Chem.* 244 6049–6055. 5389100

[B47] Mendes R. Garbeva P. Raaijmakers J. M. (2013). The rhizosphere microbiome: significance of plant beneficial, plant pathogenic, and human pathogenic microorganisms. *FEMS Microbiol. Rev.* 375 634–663. 10.1111/1574-6976.12028 23790204

[B48] Mhadhbi H. Jebara M. Limam F. Aouani M. (2004). Rhizobial strain involvement in plant growth, nodule protein compositionand antioxidant enzyme activities of chickpea-rhizobia symbioses: modulation by salt stress. *Plant Physiol. Biochem.* 42 717–722. 10.1016/j.plaphy.2004.07.005 15474377

[B49] Mittler R. (2002). Oxidative stress, antioxidants and stress tolerance. *Trends Plant Sci.* 7 405–410. 10.1016/s1360-1385(02)02312-9 12234732

[B50] Naumova N. Kuznetsova G. V. Alikina T. Y. Kabilov M. (2015). Bacterial 16S DNA diversity in the rhizosphere soil of the two pine species. *Biomics* 7 128–137.

[B51] Nguyen N. H. Bruns T. D. (2015). The Microbiome of *Pinus muricata* ectomycorrhizae: community assemblages, fungal species effects, and *Burkholderia* as important bacteria in multipartnered symbioses. *Microb. Ecol.* 69 914–921. 10.1007/s00248-015-0574-y 25687126

[B52] Oliva J. Suz L. Colinas C. (2009). Ecology of *Armillaria species* on silver fir (*Abies alba*) in the Spanish Pyrenees. *Ann. Forest Sci.* 66:603 10.1051/forest/2009046

[B53] Oliveros J. C. (2006). *VENNY. An Interactive Tool for Comparing Lists with Venn Diagrams.* Available at: http://bioinfogp.cnb.csic.es/tools/venny/index.html (accessed February 15, 2017).

[B54] Oros-Ortega I. Andrade-Torres A. Lara-Pérez L. A. Guzmán-Olmos R. F. Casanova-Lugo F. Sáenz-Carbonell L. A. (2017). Ectomycorrhizal ecology, biotechnology and taxonomy for the conservation and use of *Abies religiosa* in temperate areas of Mexico. *Revista Chapingo Serie Ciencias Forestales y del Ambiente* 233 411–426. 10.5154/r.rchscfa.2016.11.060

[B55] Paparu P. Dubois T. Gold C. S. Niere B. Adipala E. Coyne D. (2008). Screenhouse and field persistence of nonpathogenic endophytic *Fusarium oxysporum* in Musa tissue culture plants. *Microb. Ecol.* 55 561–568. 10.1007/s00248-007-9301-7 18058162

[B56] Plett J. M. Martin F. M. (2018). Know your enemy, embrace your friend: using omics to understand how plants respond differently to pathogenic and mutualistic microorganisms. *Plant J.* 93 729–746. 10.1111/tpj.13802 29265527

[B57] Polle A. Otter T. Seifert F. (1994). Apoplastic peroxidases and lignification in needles of Norway spruce *Picea abies* L. *Plant Physiol.* 106 53–60. 10.1104/pp.106.1.53 12232302 PMC159498

[B58] Proença D. N. Romeu F. Kublik S. Schöler A. Vestergaard G. Schloter M. (2016). The microbiome of endophytic, wood colonizing bacteria from pine trees as affected by pine wilt disease. *Sci. Rep.* 7:4205. 10.1038/s41598-017-04141-6 28646202 PMC5482821

[B59] Rasmussen S. Barah P. Suarez-Rodriguez M. C. Bressendorff S. Friis P. Costantino P. (2013). Transcriptome responses to combinations of stresses on *Arabidopsis*. *Plant Physiol.* 161 1783–1794. 10.1104/pp.112.210773 23447525 PMC3613455

[B60] Rudawska M. Pietras M. Smutek I. Strzeliński P. Leski T. (2016). Ectomycorrhizal fungal assemblages of *Abies alba* Mill. outside its native range in Poland. *Mycorrhiza* 261 57–65. 10.1007/s00572-015-0646-3 26071873 PMC4700082

[B61] Sandhya V. Ali S. Z. Grover M. Reddy G. Venkateswaralu B. (2010). Effect of plant growth promoting Pseudomonas spp. on compatible solutes, antioxidant status and plant growth of maize under drought stress. *Plant Growth Regul.* 62 21–30.

[B62] Sarkar J. Chakraborty B. Chakraborty U. (2018). Plant growth promoting rhizobacteria protect wheat plants against temperature stress through antioxidant signalling and reducing chloroplast and membrane injury. *J. Plant Growth Regul.* 37 1396–1412. 10.1007/s00344-018-9789-8

[B63] Schreiter S. Ding G. C. Heuer H. Neumann G. Sandmann M. Grosch R. (2014). Effect of the soil type on the microbiome in the rhizosphere of field-grown lettuce. *Front. Microbiol.* 5:144. 10.3389/fmicb.2014.00144 24782839 PMC3986527

[B64] Shi C. Wang C. Xu X. Huang B. Wu L. Yang D. (2015). Comparison of bacterial communities in soil between nematode-infected and nematode-uninfected *Pinus massoniana* pinewood forest. *Appl. Soil Ecol.* 85 11–20. 10.1016/j.apsoil.2014.08.008

[B65] Stenstrom S. Ndobe N. Jonsson M. Stenlid J. Menkis A. (2014). Root-Associated fungi of healthy- looking *Pinus sylvestris* and *Picea abies* seedelings in Swedish forest nurseries. *Scand. J. Forest Res.* 29 12–21.

[B66] Stokholm M. S. Wulff E. G. Zida P. E. Ibié G. T. Néya B. J. Soalla W. R. (2016). DNA barcoding and isolation of vertically transmitted ascomycetes in sorghum from Burkina Faso: *Epicoccum sorghinum* is dominant in seedlings and appears as a common root pathogen. *Microbiol. Res.* 191 38–50. 10.1016/j.micres.2016.05.004 27524652

[B67] Suna H. Terhonena E. Koskinenb K. Paulin L. Kasanen R. Asiegbua F. O. (2014). Bacterial diversity and community structure along different peat soils in boreal forest. *Appl. Soil Ecol.* 74 37–45. 10.1016/j.apsoil.2013.09.010

[B68] Sundberg C. Al-Soud W. A. Larsson M. Alm E. Yekta S. S. Svensson B. H. (2013). 454 pyrosequencing analyses of bacterial and archaeal richness in 21 full-scale biogas digesters. *FEMS Microbiol. Ecol.* 85 612–626. 10.1111/1574-6941.12148 23678985

[B69] Taylor A. F. S. Martin F. Read D. J. (2000). “Fungal Diversity in Ectomycorrhizal Communities of Norway Spruce [Picea abies L. Karst.] and Beech Fagus sylvatica L. Along North-South Transects in Europe,” in *Carbon and Nitrogen Cycling in European Forest Ecosystems. Ecological Studies Analysis and Synthesis*, Vol. 142 ed. Schulze E. D. (Berlin: Springer), 343–365. 10.1007/978-3-642-57219-7_16

[B70] Tedersoo L. Smith E. M. (2013). Lineages of ectomycorrhizal fungi revisited: foraging strategies and novel lineages revealed by sequences from belowground. *Fungal Biol. Rev.* 27 83–99. 10.1016/j.fbr.2013.09.001

[B71] Torsvik V. Øvreås L. Thingstad T. F. (2002). Prokaryotic diversity-magnitude, dynamics, and controlling factors. *Science* 296 1064–1066. 10.1126/science.1071698 12004116

[B72] Uroz S. Buée M. Deveau A. Mieszkin S. Martin F. (2016). Ecology of the forest microbiome: Highlights of temperate and boreal ecosystems. *Soil Biol. Biochem.* 103 471–488. 10.1016/j.soilbio.2016.09.006

[B73] Vanderkoornhuyse P. Quaiser A. Duhamel M. Le Van D. dufresne A. (2015). The importance of the microbiome of the plant holobiont. *New Phytol.* 206 1196–1206. 10.1111/nph.13312 25655016

[B74] Vardharajula S. Zulfikar Ali S. Grover M. Reddy G. Bandi V. (2011). Drought-tolerant plant growth promoting *Bacillus spp*., effect on growth, osmolytes, and antioxidant status of maize under drought stress. *J. Plant Interact.* 6 1–14. 10.1080/17429145.2010.535178

[B75] Vrålstad T. Holst-Jensen A. Schumacher T. (1998). The postfire *Discomycete Geopyxis carbonaria* Ascomycota is a biotrophic root associate with Norwayspruce *Picea abies* in nature. *Mol. Ecol.* 7 609–616. 10.1046/j.1365-294x.1998.00365.x 9633103

[B76] Vu T. Hauschild R. Sikora R. A. (2006). *Fusarium oxysporum* endophytes induced systemic resistance against Radopholus similis on banana. *Nematology* 8 847–852. 10.1163/156854106779799259

[B77] Vujanovic V. Hamelin C. Bernier L. Vujanovic G. St-Arnaud M. (2007). Fungal diversity, dominance, and community structure in the rhizosphere of clonal *Picea mariana* plants throughout nursery production chronosequences. *Microb. Ecol.* 54 672–684. 10.1007/s00248-007-9226-1 17347891

[B78] Vurukonda S. Vardharajula S. Shrivastava M. SkZ A. (2016). Enhancement of drought stress tolerance in crops by plant growth promoting rhizobacteria. *Microbiol. Res.* 184 13–24. 10.1016/j.micres.2015.12.003 26856449

[B79] Wagner M. R. Lundberg D. S. del Rio T. G. Tringe S. G. Dangl J. L. Mitchell-Olds T. (2016). Host genotype and age shape the leaf and root microbiomes of a wild perennial plant. *Nat. Commun.* 7 1–15. 10.1038/ncomms12151 27402057 PMC4945892

[B80] Warton D. I. Wright S. T. Wang Y. (2012). Distance-based multivariate analyses confound location and dispersion effects. *Methods Ecol. Evol.* 3 89–101. 10.1111/j.2041-210X.2011.00127.x

[B81] Ważny R. (2014). Ectomycorrhizal communities associated with silver fir seedlings *Abies alba* Mill. differ largely in mature silver fir stands and in Scots pine forecrops. *Ann. Forest Sci.* 71 801–810. 10.1007/s13595-014-0378-0

[B82] Wu Z. Hao Z. Zeng Y. Guo L. Huang L. Chen B. (2015). Molecular characterization of microbial communities in the rhizosphere soils and roots of diseased and healthy *Panax notoginseng*. *Antonie van Leeuwenhoek* 1085 1059–1074. 10.1007/s10482-015-0560-x 26296378

[B83] Yeoh Y. Dennis P. Paungfoo-Lonhienne C. Weber L. Brackin R. Ragan M. (2017). Evolutionary conservation of a core root microbiome across plant phyla along a tropical soil chronosequence. *Nat. Commun.* 8:215. 10.1038/s41467-017-00262-8 28790312 PMC5548757

[B84] Yilmaz P. Parfrey L. W. Yarza P. Gerken J. Pruesse E. Quast C. (2014). The SILVA and all-species Living Tree Project (LTP) taxonomic frameworks. *Nucleic Acids Res.* 42 643–648. 10.1093/nar/gkt1209 24293649 PMC3965112

[B85] Yoshimura K. Yabuta Y. Ishikawa T. Shigeoka S. (2000). Expression of spinach ascorbate peroxidase isoenzymes in response to oxidative stresses. *Plant Physiol.* 123 223–234. 10.1104/pp.123.1.223 10806239 PMC58996

[B86] Yu H. X. Wang C. Y. Tang M. (2013). Fungal and bacterial communities in the rhizosphere of *Pinus tabulaeformis* related to the restoration of plantations and natural secondary forests in the Loess Plateau, northwest China. *Sci. World J.* 2013 1–12. 10.1155/2013/606480 24459438 PMC3886228

[B87] Zhalnina K. B. Louie Z. Hao N. Mansoori U. N. da Rocha S. Shi H. (2018). Dynamic root exudate chemistry and microbial substrate preferences drive patterns in rhizosphere microbial community assembly. *Nat. Microbiolo.* 3 470–480. 10.1038/s41564-018-0129-3 29556109

[B88] Zhu J. (2001). Plant salt tolerance. *Trends Plant Sci.* 6 66–71. 11173290 10.1016/s1360-1385(00)01838-0

[B89] Zulueta-Rodriguez R. Hernandez-Montiel L. G. Murillo-Amador B. Rueda-Puente E. O. Capistron L. L. Troyo-Domiguez E. (2015). Effect of hydropriming and biopriming on seed germination and growth of two Mexican fir tree species in danger of extinction. *Forests* 69 3109–3122. 10.3390/f6093109

