# Supplementary material for: Root-Associated Microbial Communities of Abies nordmanniana: Insights Into Interactions of Microbial Communities With Antioxidative Enzymes and Plant Growth
Source: Front Microbiol. 2019 Aug 22;10:1937. doi: 10.3389/fmicb.2019.01937 (PMC6714061; doi:10.3389/fmicb.2019.01937)
Supplement: Supplementary file 1 [file Data_Sheet_1.PDF]

Supplementary Material

Supplementary Tables

S Table 1. Climatic conditions at the sampling sites located in Germany and Denmark. The climatic data were obtained from the German National meteorological Service (http://www.dwd.de) and the Danish National meteorological Service (http://www.dmi.dk).

	**Location**	
	**Hohenlocksted, Schleswig-Holstein, Germany**	**Hadsund, Mariagerfjord, Denmark**
Sampling date	12/07/2016	26/07/2016
Temperature max. (May- July 2016)	18 ˚C - 21 ˚C	25 ˚C - 28 ˚C
Temperature min. (May- July 2016)	2 ˚C - 12 ˚C	0.4 ˚C - 9 ˚C
Average temperature (May-July 2016)	17 ˚C	16 ˚C
Rain (May-July)	21-101 mm	41-80 mm
Hours of sun (May- July 2016)	275-176 hrs.	259-152 hrs.
**Historical data (3 years)**		
Yearly average temperature max. (2014-2016)	29 ˚C	29 ˚C
Yearly average temperature min. (2014-2016)	-12 ˚C	-10 ˚C
Yearly rain (2014-2016)	801 mm	932 mm
Yearly hours of sun (2014-2016)	1664 hrs.	1637 hrs.

S Table 2. Size (cm) of each sampled plant of *A. nordmanniana* collected at both sampling sites. Plant heights were measured from the top of the apical bud to the end of the main root.

	**Plant height (cm), at the two sampling sites**	
**small plants**	**Germany**	**Denmark**
Sample 1	21	22
Sample 2	28	24
Sample 3	25	27
Sample 4	28	28
Sample 5	23	25
**Average height small plants**	**25**	**25**
**Tall plants**		
Sample 1	55	63
Sample 2	63	61
Sample 3	61	59
Sample 4	61	57
Sample 5	59	60
**Average height tall plants**	**60**	**60**

S Table 3. Bulk soil composition from site 1; Baumschule Engler nursery, Germany and site 2; Primo Plant Ejendomme ApS nursery, Denmark, soil analysis was performed by Eurofins Agro Testing Denmark A/S (www.eurofins.dk).

**Test (soil pack for Christmas tree)**	**Site 1, Germany**	**Site 2, Denmark**
Sulfur in Sulphate-ICP-OES	<10 mg/kg DM.	<10 mg/kg DM.
Manganese (Mn)- ICP-OES	9.0 mg/kg DM.	3.0 mg/kg DM.
Boron (B)- ICP-OES	0.71 mg/kg DM.	0.34 mg/kg DM.
Reaction Number-Potentiometry	5	6.2
Phosphorus (P)-Spectroscopy (FIA)	9.4 mg/100 g DM.	7.4 mg/100 g DM.
Potassium (K) (available for plant)-Spectroscopy (FIA)	4.3 mg/100 g DM.	6.8 mg/100 g DM.
Magnesium (Mg) (available for plant)-ICP-OES	3.3 mg/100 g DM.	6.5 mg/100 g DM.
Sodium (Na) (available for Plant)-ICP-OES	1.3 mg/100 g DM.	1.4 mg/100 g DM.
Copper (Cu)-ICP-OES	1.4 mg/kg DM.	2.1 mg/kg DM.
Iron (Fe)- ICP-OES	72 mg/kg DM.	110 mg/kg DM.
Zinc (Zn)-ICP-OES	1.7 mg/kg DM.	1.1 mg/kg DM.
Nitrogen-ISO 13878 / Dumas method	0.06%	0.06%
Organic matter	1.00%	1.30%

ICP-OES= Inductively Coupled Plasma – Optical Emission Spectrometry; FIA= Flow Injection Analysis- spectrometry; DM= Dry Matter.

S Table 4. MiSeq Illumina fusion primers for amplicon sequencing of eukaryotic 18S rDNA from rhizosphere samples of *A. nordmanniana*.

ABIES sample ID	Forward tagged fusion primers					Reverse tagged fusion primers					
	**MiSeq Illumina Fw Linker**	**Internal barcode**	**2xN**	**Heterogeneity spacer bp[a]**	**18S-F2**	**MiSeq Illumina Rv Linker**	**Internal barcode**	**2xN**	**12 bp insert**	**18S-R2aeh**	**18S-R2g[b]**
**ABIES-01**		ACGAGTGCGT					ACGAGTGCGT				
**ABIES-02**		ACGCTCGACA		T			ACGCTCGACA				
**ABIES-03**		AGCACTGTAG		GT			AGCACTGTAG				
**ABIES-04**		ATCAGACACG		CGA			ATCAGACACG				
**ABIES-05**		ATATCGCGAG		ATGA			ATATCGCGAG				
**ABIES-06**		CGTGTCTCTA		TGCGA			CGTGTCTCTA				
**ABIES-07**		CTCGCGTGTC		GAGTGG			CTCGCGTGTC				
**ABIES-08**		TCTCTATGCG		CCTGTGG			TCTCTATGCG				
**ABIES-09**		TGATACGTCT					TGATACGTCT				
**ABIES-10**		CATAGTAGTG		T			CATAGTAGTG				
**ABIES-11**		CGAGAGATAC		GT			CGAGAGATAC				
**ABIES-12**		ATACGACGTA		CGA			ATACGACGTA				
**ABIES-13**		TCACGTACTA		ATGA			TCACGTACTA				
**ABIES-14**	5'TCGTCGGCAG	CGTCTAGTAC		TGCGA		5'GTCTCGTGGG	CGTCTAGTAC				
**ABIES-15**	CGTCAGATGTG	TCTACGTAGC	NN	GAGTGG	AGGATTGA	CTCGGAGATGT	TCTACGTAGC	NN	AGTGCG	GKCAGGG	CGTAGTC
**ABIES-16**	TATAAGAGACA	TGTACTACTC		CCTGTGG	CAGATTGA'	GTATAAGAGAC	TGTACTACTC		TCTAAC	ACGTAWT	AACGCAA
**ABIES-17**	G	ACGACTACAG			3	AG	ACGACTACAG			C'3	G'3
**ABIES-18**		CGTAGACTAG		T			CGTAGACTAG				
**ABIES-19**		TACGAGTATG		GT			TACGAGTATG				
**ABIES-20**		TACTCTCGTG		CGA			TACTCTCGTG				
**ABIES-21**		TAGAGACGAC		ATGA			TAGAGACGAG				
**ABIES-22**		TCGTCGCTCG		TGCGA			TCGTCGCTCG				
**ABIES-23**		ACATACGCGT		GAGTGG			ACATACGCGT				
**ABIES-24**		ACGCGAGTAT		CCTGTGG			ACGCGAGTAT				
**ABIES-25**		ACTACTATGT					ACTACTATGT				
**ABIES-26**		ACTGTACAGT		T			ACTGTACAGT				
**ABIES-27**		AGACTATACT		GT			AGACTATACT				
**ABIES-28**		AGCGTCGTCT		CGA			AGCGTCGTCT				
**ABIES-29**		AGTACGCTAT		ATGA			AGTACGCTAT				
**ABIES-30**		ATAGAGTACT		TGCGA			ATAGAGTACT				

[a] Heterogeneity spacer derived from Fadrosh *et al.*, 2014. [b] as for the 18S-R2aeh, the 18S-R2g reverse primer is also fused to the Rv linker, the different internal barcodes, the 2xN and the 12 bp insert.

S Table 5. Dissimilarity analysis of the microbial community in the bulk soil and rhizosphere of tall and small plants *A. nordmanniana* collected from two sampling sites.

**Sampling Groups (bacterial communities)**	**Adonis test**		
	**F**	**R^2**	***P***
Sampling site[1.]	59.171	0.134	0.001 ***
Plant size[2.]	11.331	0.028	0.275
**Sampling Groups (fungal communities)**			
Sampling site[1.]	30.596	0.145	0.007 **
Plant size[2.]	0.865	0.045	0.529

Significance codes: 0 '***' 0.001 '**' 0.01 '*' 0.05 '.' 0.1 ' ' 1

[1]. Site: Germany, Denmark. [2]. Plant size: tall plants, small plants

S Table 6. Percent contribution of rhizosphere bacteria (order level), contributing with 64% to sample site dissimilarity. Values based on SIMPER analysis of the Bray-Curtis dissimilarity distance.

**Bacterial taxa**	**Contribution %**
Burkholderiales	12.58
Rhizobiales	10.8
Acidobacteriales	7.77
Actinomycetales	7.59
Xanthomonadales	7.24
Rickettsiales	6.07
Rhodospirillales	5.188
Sphingobacteriales	3.45
Saprospirales	3.03

S Table 7. Percent contribution of rhizosphere fungi (order level), contributing with 94% to sample site and plant size dissimilarity. Values based on SIMPER analysis of the Bray-Curtis dissimilarity distance.

**Fungal taxa**	**Contribution %**
Hypocreales	29.42
Agaricales	28.47
Pezizales	23.07
Helotiales	5.78
Trechisporales	2.33
Xylariales	2.32
Mortierellales	2.22

S Table 8. Percent contribution of rhizosphere fungal genera, contributing with 88% to sample site and plant size dissimilarity. Values based on SIMPER analysis of the Bray-Curtis dissimilarity distance.

**Genus**	**Contribution %**
Fusarium	21.29
Trechispora	17.88
Cystofilobasidium	12.56
Hyaloscypha	11.10
Sporobolomyces	9.17
Sporothrix	8.45
Trichoderma	7.84
Umbelopsis	5.51

Supplementary Figures

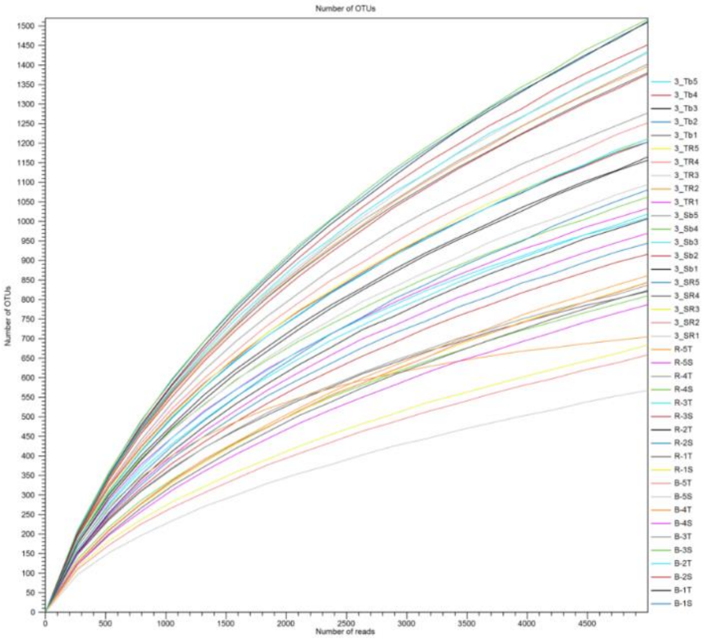

Supplementary Figure 1. Rarefaction curves with the observed bacterial OTUs from the rhizosphere and bulk soil of tall and small plants of *A. nordmanniana* collected in two different sampling sites. *Legends in the graphic starting with the number 3_ belong to samples collected in Denmark. B, b=bulk soil; R=rhizosphere; T=tall plants; S= small plants, each number is the sample number.

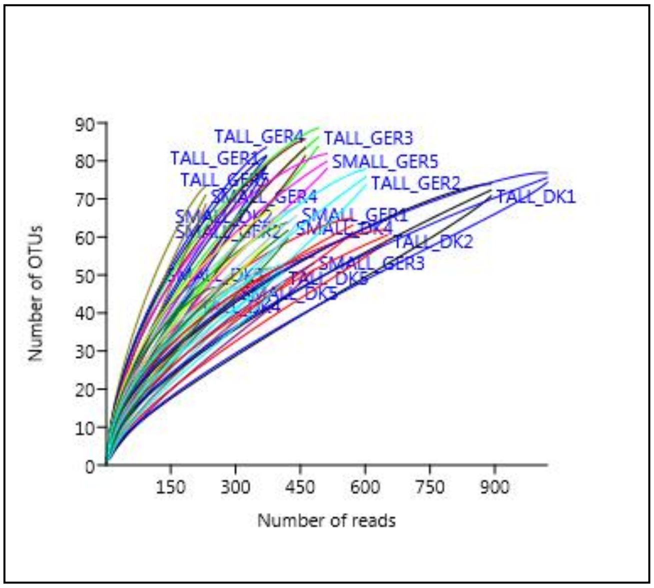

Supplementary Figure 2. Rarefaction curves with the observed fungal OTUs from the rhizosphere of tall and small plants of *A. nordmanniana* collected in two different sampling sites.

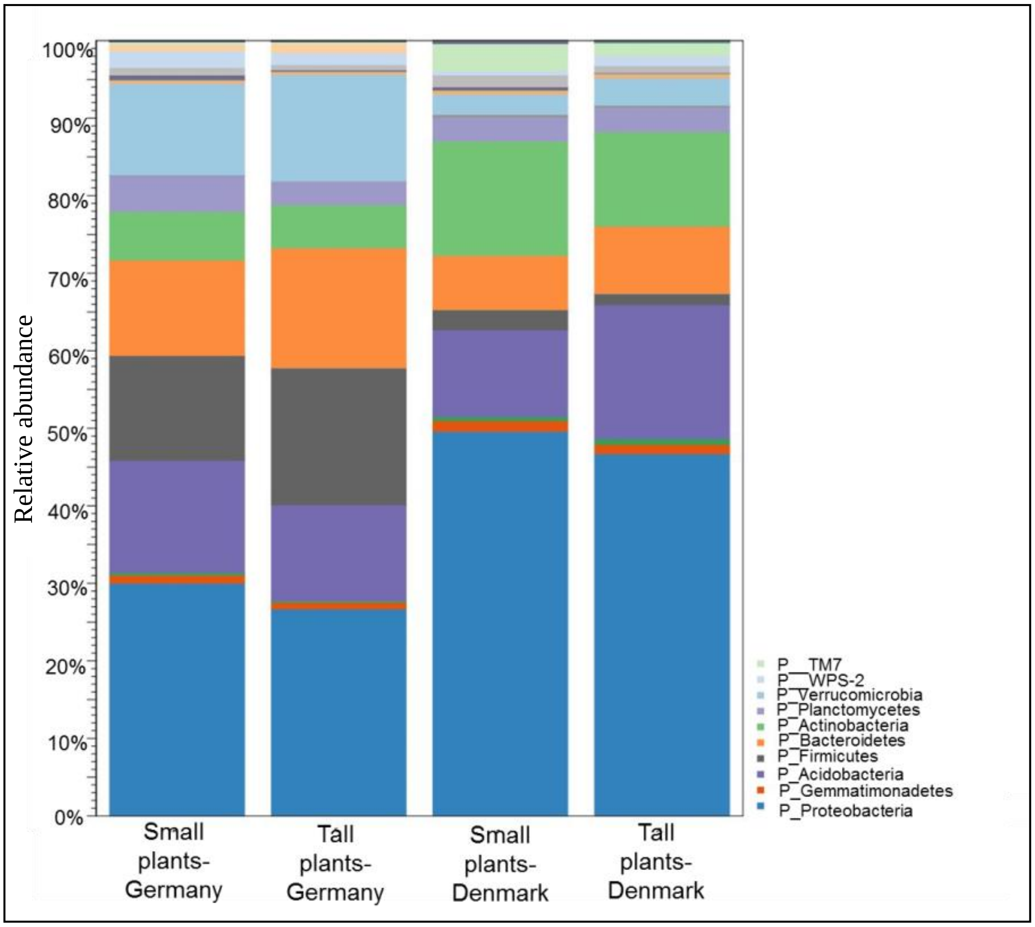

Supplementary Figure 3. Stacked bar plot showing OTU-based relative abundance of bacterial phyla in the rhizosphere of 3-year-old *A. nordmanniana.* Analysis was based on 16S rRNA gene sequences. Capital letter P_: indicates phylum level.

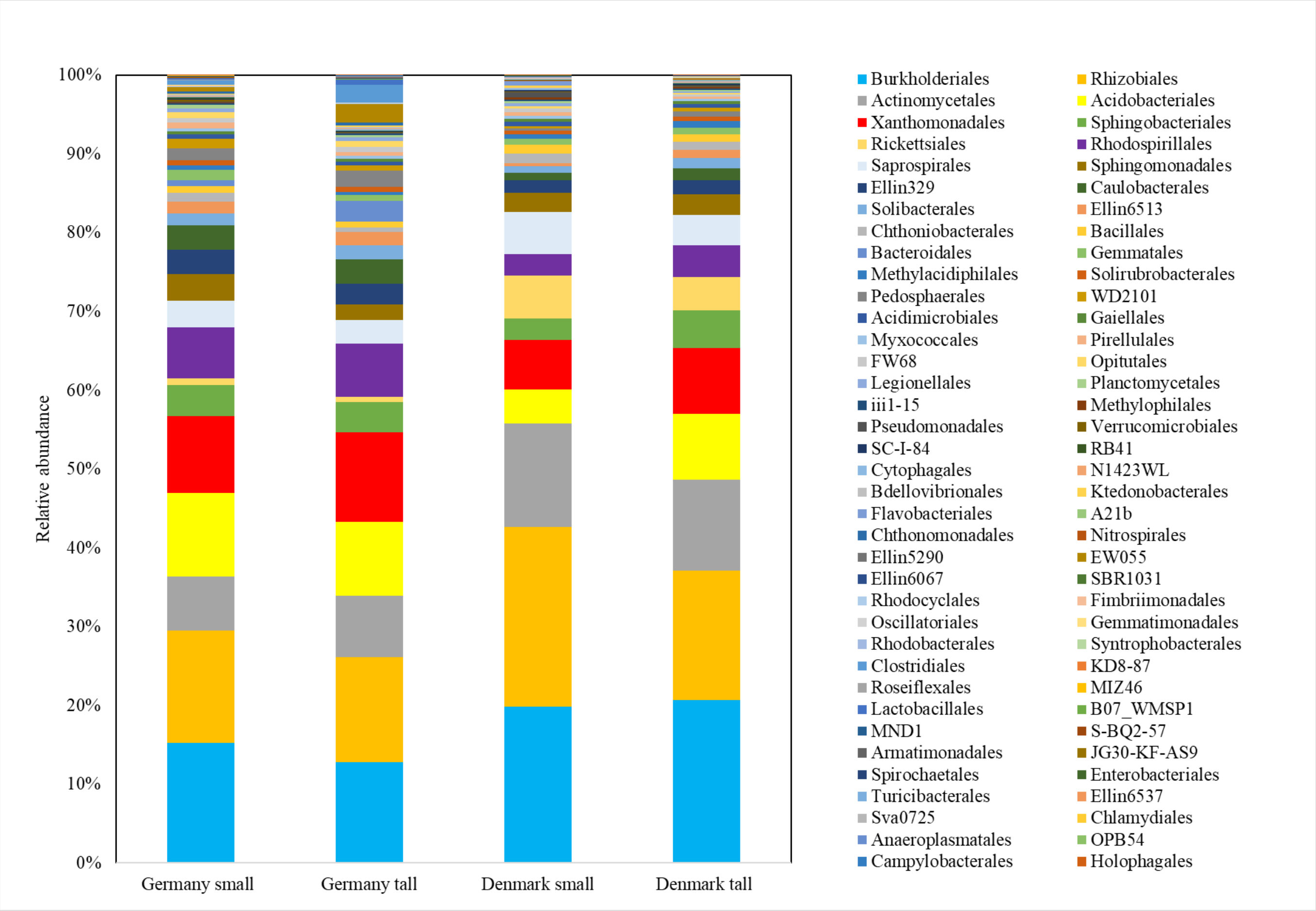

Supplementary Figure 4. Stacked bar plot of the shared rhizosphere bacterial (order level) relative abundance, between both sampling sites and plant sizes of *A. nordmanniana*. Bar plot was constructed using the list of shared OTUs from the Venn diagram, and the mean relative abundances of the five biological samples for each plant size and sampling site.

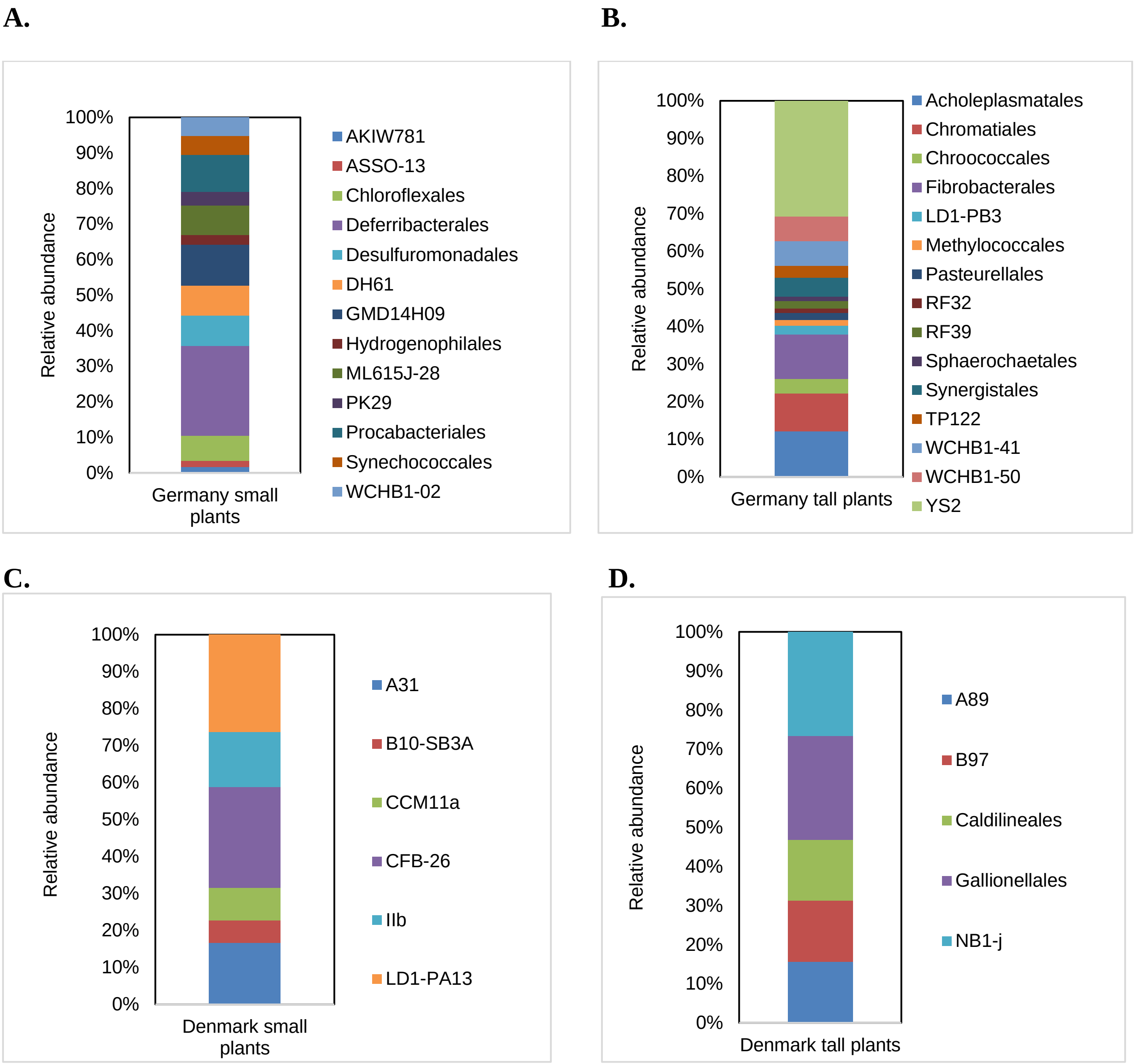

Supplementary Figure 5. Stacked bar plot showing the unique rhizosphere bacterial orders in each sampling site and plant size of *A. nordmanniana.* **(A).** Small plants from Germany. **(B).** Tall plants from Germany. **(C).** Small plants from Denmark. **(D).** Tall plants from Denmark. Bar plot was constructed using the list of unique OTUs from the Venn diagram, and the mean relative abundances of the five biological samples for each plant size and sampling site.

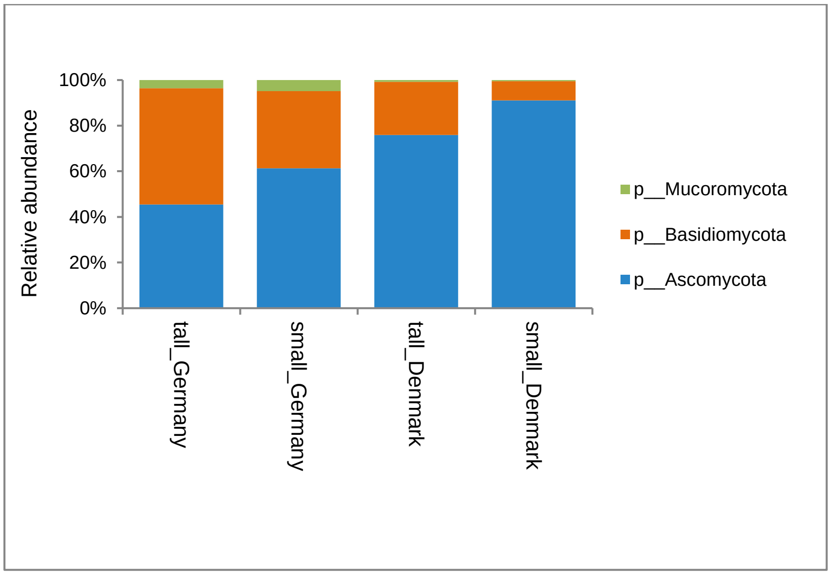

Supplementary Figure 6. Stacked bar plot showing OTU relative sequence abundance of fungal phyla in the rhizosphere of tall and small *A. nordmanniana* plants from both sampling sites. Analysis based on 18S rRNA gene sequences. Capital letter P_: indicates phylum level.

A.

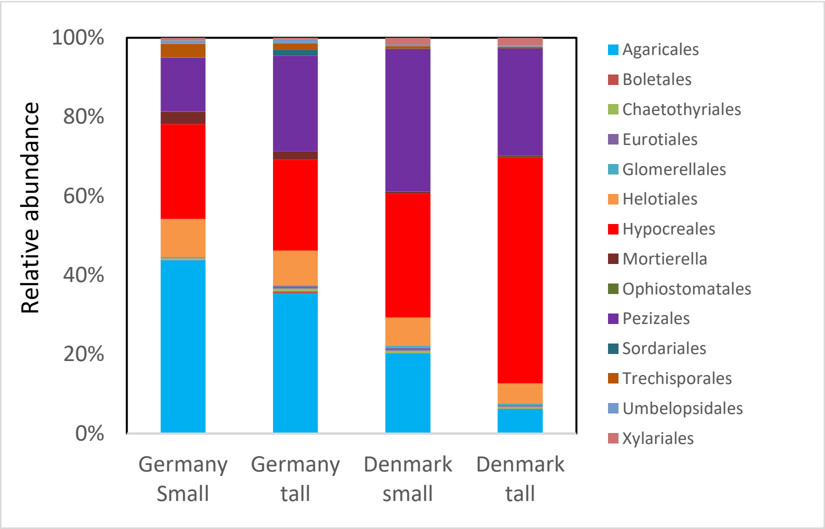

B.

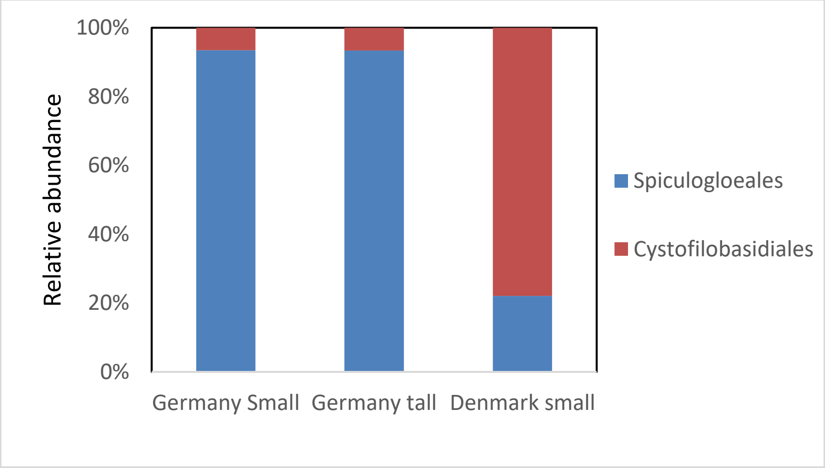

Supplementary Figure 7. Stacked bar plot showing the relative abundance of shared rhizosphere fungal OTUs at the order level. **(A).** Between both sampling sites and plant sizes of *A. nordmanniana*. **(B).** Between German samples from tall and small plants and Danish samples small plants. Bar plots were constructed using the list of shared OTUs from the Venn diagram result and the mean relative abundances of the five biological samples for each plant size and sampling site.
